# A comparison of automated atrophy measures across the frontotemporal dementia spectrum: Implications for trials

**DOI:** 10.1016/j.nicl.2021.102842

**Published:** 2021-10-05

**Authors:** Elizabeth Gordon, Martina Bocchetta, Jennifer Nicholas, David M Cash, Jonathan D Rohrer

**Affiliations:** a Dementia Research Centre, Department of Neurodegenerative Disease, UCL Queen Square Institute of Neurology, London, UK; b Department of Medical Statistics, London School of Hygiene and Tropical Medicine, London, UK; c The UK Dementia Research Institute (UK DRI), London, UK

**Keywords:** Frontotemporal dementia, Magnetic resonance imaging, Automated segmentation, Longitudinal atrophy, Neuroimaging biomarkers, Volumetric imaging, Clinical trials

## Abstract

**Background:**

Frontotemporal dementia (FTD) is a common cause of young onset dementia, and whilst there are currently no treatments, there are several promising candidates in development and early phase trials. Comprehensive investigations of neuroimaging markers of disease progression across the full spectrum of FTD disorders are lacking and urgently needed to facilitate these trials.

**Objective:**

To investigate the comparative performance of multiple automated segmentation and registration pipelines used to quantify longitudinal whole-brain atrophy across the clinical, genetic and pathological subgroups of FTD, in order to inform upcoming trials about suitable neuroimaging-based endpoints.

**Methods:**

Seventeen fully automated techniques for extracting whole-brain atrophy measures were applied and directly compared in a cohort of 226 participants who had undergone longitudinal structural 3D T1-weighted imaging. Clinical diagnoses were behavioural variant FTD (n = 56) and primary progressive aphasia (PPA, n = 104), comprising semantic variant PPA (n = 38), non-fluent variant PPA (n = 42), logopenic variant PPA (n = 18), and PPA-not otherwise specified (n = 6). 49 of these patients had either a known pathogenic mutation or postmortem confirmation of their underlying pathology. 66 healthy controls were included for comparison. Sample size estimates to detect a 30% reduction in atrophy (80% power; 0.05 significance) were computed to explore the relative feasibility of these brain measures as surrogate markers of disease progression and their ability to detect putative disease-modifying treatment effects.

**Results:**

Multiple automated techniques showed great promise, detecting significantly increased rates of whole-brain atrophy (*p*<0.001) and requiring sample sizes of substantially less than 100 patients per treatment arm. Across the different FTD subgroups, direct measures of volume change consistently outperformed their indirect counterparts, irrespective of the initial segmentation quality. Significant differences in performance were found between both techniques and patient subgroups, highlighting the importance of informed biomarker choice based on the patient population of interest.

**Conclusion:**

This work expands current knowledge and builds on the limited longitudinal investigations currently available in FTD, as well as providing valuable information about the potential of fully automated neuroimaging biomarkers for sporadic and genetic FTD trials.

## 1 Introduction

The term ‘frontotemporal dementia’ (FTD) refers to a heterogeneous spectrum of clinically, genetically and pathologically diverse neurodegenerative disorders. Clinically, people present with either predominant changes in behaviour, social conduct and personality (behavioural variant, bvFTD), or language impairment (primary progressive aphasia, PPA). PPA can be further divided into three main subtypes, semantic variant (svPPA), non-fluent variant (nfvPPA) and logopenic variant (lvPPA) (Gorno-Tempini et al., 2011). In addition, a subgroup of people that do not fulfil the current diagnostic criteria for the three canonical PPA subtypes is recognised, referred to here as PPA not otherwise specified (PPA-NOS) (Marshall et al., 2018, Rohrer et al., 2010b). Genetically, FTD is a highly heritable set of disorders, with approximately a third of patients exhibiting an autosomal dominant form (Greaves and Rohrer, 2019, Rohrer and Warren, 2011). Mutations in the microtubule-associated protein tau (*MAPT*) and progranulin (*GRN*) genes or a hexanucleotide expansion in the chromosome 9 open reading frame 72 (*C9orf72*) gene are the major contributors (Rohrer et al., 2009, Warren et al., 2013). Pathologically, neuronal inclusions containing abnormal forms of tau or TAR DNA-binding protein 43 (TDP-43) are the key forms associated with FTD, with the latter classified into four subtypes TDP-43 Type A – D (Lashley et al., 2015, Mackenzie et al., 2011, Mackenzie et al., 2010, Seelaar et al., 2010).

Whilst there are no treatments for FTD currently available, there are a number of candidate molecules demonstrating the promise of disease modification. In preparation for these trials, it is crucial to find accurate and sensitive biomarkers to evaluate the efficacy of these interventions and to help inform trial design so that they are powered to detect a clinically relevant change in these markers. Methods for quantifying brain atrophy rates from serial magnetic resonance imaging (MRI) are already employed as surrogate non-invasive markers in other neurodegenerative intervention trials (Cash et al., 2014, Tabrizi et al., 2019, Zeun et al., 2019), but have yet to be studied extensively in FTD. Incorporating imaging biomarkers as surrogate end-points may allow detection of disease-modifying effects with fewer participants than standard cognitive tests (Schott et al., 2010, Whitwell et al., 2015b) and have the potential for detecting these changes presymptomatically in genetic FTD (Borroni et al., 2008, Cash et al., 2018, Dopper et al., 2013, Lee et al., 2017, Panman et al., 2019, Papma et al., 2017, Popuri et al., 2018, Rohrer et al., 2015). Therefore, validation within the different FTD subgroups is important to better ascertain their utility as we move towards treatments directly targeting FTD-associated pathologies.

As a potential trial biomarker, longitudinal neuroimaging measures need to be indicative of the underlying pathological process, be associated with disease progression and sensitive to small changes given initial treatment effects are likely to be subtle. In practical terms, they should be relatively quick and easy to apply as well as consistently and accurately measure change over serial images. Importantly, they should be reliable across different centres and image acquisitions because large trials of a rare condition like FTD will require multicentre or international collaborations to recruit a sufficient number of individuals. A helpful metric to assess the utility of a potential biomarker is to calculate the sample size required to detect a treatment effect with sufficient statistical power. Good biomarkers produce lower sample sizes required to power a trial for shorter intervals, which would ultimately prove time- and cost-effective. Whilst longitudinal neuroimaging investigations are relatively scarce in FTD, several studies have investigated global volumetric change measures and reported associated sample size calculations (Gordon et al., 2010, Knopman et al., 2009, Mahoney et al., 2015, Pankov et al., 2016, Rohrer et al., 2012, Rohrer et al., 2008, Whitwell et al., 2015a, Staffaroni et al., 2019: summarised in Supplementary Table S1). The variability and inconsistency in these sample size estimates is likely due to small study cohorts capturing heterogenous patient subsets within the broad FTD spectrum as well as differing segmentation protocols and accuracy between studies.

The labour-intensive nature of manual delineation and the issue of intra-rater variability limits its feasibility for application in large cohorts or extensive multicentre trials, and therefore considerable attention has been paid to the development of increasingly sophisticated methods of automated segmentation (González-Villà et al., 2016, Iglesias and Sabuncu, 2015, Shaikh and Ali, 2019). There are currently multiple freely available tools that are widely used in clinical research (Ashburner and Friston, 2005, Cardoso et al., 2015, Fischl et al., 2002, Ledig et al., 2015, Leung et al., 2011, Zhang et al., 2001). Previous comparisons have tended to focus on the most commonly used fully integrated packages and pipelines, such as those included in Freesurfer, Statistical parametric mapping (SPM) and the FSL library (Johnson et al., 2017, Katuwal et al., 2016).

Whilst there are different approaches and pipelines, they generally involve brain extraction (identifying brain from non-brain voxels on the scan); tissue classification (identifying grey matter (GM)/white matter (WM) and cerebrospinal fluid (CSF)); intensity correction (to account for image inhomogeneity); spatial normalisation or registration (to match to a template or atlas) and final labelling of regions of interest (ROIs) onto the unsegmented target image. Although automated segmentation techniques show great promise for measuring disease progression and may allow detection of disease-related treatment effects, it remains unclear as to which available methodologies are well-suited for FTD. Therefore, this study aims to investigate automated brain volumetry and global atrophy measures across the FTD spectrum, by applying a range of automated atrophy measures to a single, large FTD cohort. More specifically, it focuses on the direct head-to-head comparative performance of these techniques in providing accurate longitudinal measures for potential non-invasive biomarkers in trials, including identifying which of the measures provides the lowest feasible sample size to detect a meaningful treatment effect within each FTD clinical, genetic and pathology subgroup.

## 2 Methods

### 2.1 Participants

Participants consisted of a consecutive retrospective cohort who had taken part in longitudinal FTD research studies at the University College London Dementia Research Centre (DRC), recruited through the Cognitive Disorders Clinic at the National Hospital of Neurology and Neurosurgery (NHNN) between 1992 and 2018. Cognitively healthy controls in the study were commonly partners, family members or carers of research participants. Participants were included if they had undergone at least two volumetric 3D T1-weighted MR images performed on the same scanner. Image pairs with less than 6 months interval were excluded to improve the signal-to-noise ratio of longitudinal change measurement (Schott et al., 2006). For the 46 individuals with multiple serial images, the earliest passing pair was included for analysis if, following visual assessment, no extensive differences in contrast and positioning were identified or artefacts that might preclude clear delineation of the brain boundary. If one of these images was not passable, the next earliest image was chosen to complete the pair.

These initial inclusion criteria resulted in a longitudinal neuroimaging cohort consisted of 262 individuals: 184 people with FTD and 78 healthy controls. The people with FTD had the following diagnoses: bvFTD (n = 66), svPPA (n = 45), nfvPPA (n = 45), lvPPA (n = 21), and PPA-NOS (n = 7). 34 of these had a pathogenic mutation in *MAPT* (n = 16), *C9orf72* (n = 10) or *GRN* (n = 8). An additional 19 patients had postmortem confirmation of their underlying pathology: tau, including those with *MAPT* mutations (n = 19 total), and TDP-43, including those with *C9orf72* and *GRN* mutations (n = 34 total). Supplementary Table S2 provides the full demographic summary for this cohort.

The key focus of this study is the direct comparative performance of currently available automated segmentation and longitudinal volume change measures. Therefore, this original longitudinal neuroimaging cohort was further refined following the application of all segmentation and registration techniques and both cross-sectional and longitudinal quality control (QC). This resulted in a common subset cohort (n = 226) including only individuals who had no missing values for any of the segmentation or longitudinal pipeline measures. This ensured that the direct head-to-head comparison was performed on identical datasets of images. Supplementary Table S7 summarises where a missing value was due to a QC failure or failure of the automated pipeline to complete for each of the methods. This final common dataset consisted of 66 healthy controls and 160 people with FTD (Table 1): bvFTD (n = 56), svPPA (n = 38), nfvPPA (n = 42), lvPPA (n = 18), and PPA-NOS (n = 6). 31 of these had a pathogenic mutation in *MAPT* (n = 14), *C9orf72* (n = 9) or *GRN* (n = 8). In the pathological groups there were 17 in the tau group and 32 in the TDP-43 group. This refined cohort was used for all analyses reported in the current paper and was a representative subsample of the full original (n = 262) cohort (See Supplementary Table S2–S4 for the demographic data of the full cohort (n = 262) and annual rates of change for both the full (n = 262) and refined (n = 226) cohorts respectively for comparison).

**Table 1:** Demographics for the refined FTD subgroups and controls (n = 226) with a complete set of data points across all imaging measures.

n = 226	Healthy controls	Clinical FTD subgroups					Genetic FTD subgroups			Pathology FTD subgroups	
		bvFTD	svPPA	nfvPPA	lvPPA	PPA-NOS	*MAPT*	*C9orf72*	*GRN*	Tau	TDP-43
Number of participants	66	56	38	42	18	6	14	9	8	17	32
Clinical, genetic and pathology overlap	NA	13 *MAPT*; 8 *C9orf72*; 4 *GRN*; 4 Tau; 2 TDP-43 Type A	2 Tau; 10 TDP-43 Type C	1 *C9orf72*; 2 *GRN*; 2 TDP-43 Type A	1 *MAPT*; 5 AD	2 *GRN*, 1 TDP-43 Type A	13 bvFTD; 1 lvPPA 3 Tau	8 bvFTD; 1 nfvPPA	4 bvFTD; 2 nfvPPA; 2 PPA-NOS	14 *MAPT*; 3 postmortem confirmed	9 *C9orf72*; 8 *GRN*; 13 postmortem confirmed *
Male/Female	30 / 36	**45 / 11**	20 / 18	24 / 18	**13 / 5**	4 / 2	7 / 7	**9 / 0**	3 / 5	10 / 7	21 / 11
% male	45%	**80%**	53%	57%	**72%**	67%	50%	**100%**	38%	59%	66%
Age at baseline assessment	61.7 (11.3)	61.7 (8.5)	63.8 (7.6)	**66.0 (7.1)**	**67.7 (6.9)**	62.1 (7.7)	**54.6 (7.2)**	61.6 (5.6)	61.2 (7.2)	**55.7 (7.3)**	62.4 (6.3)
Age at symptom onset (years)	NA	55.9 (8.7)	59.3 (7.8)	61.6 (6.9)	63.4 (6.9)	60.0 (8.2)	48.6 (6.0)	54.2 (7.8)	58.0 (6.4)	50.5 (7.3)	57.4 (7.3)
Disease duration at baseline (years)	NA	**5.9 (3.8)**	4.5 (1.5)	4.4 (2.0)	4.4 (1.9)	**2.1 (1.3)**	6.0 (4.0)	7.4 (4.4)	**3.2 (3.4)**	5.2 (3.8)	5.0 (3.5)
Scan interval (years)	1.5 (0.8)	1.4 (0.8)	1.6 (0.9)	1.4 (0.6)	1.3 (0.7)	1.6 (0.8)	2.0 (1.3)	1.1 (0.3)	1.2 (0.4)	2.0 (1.2)	1.5 (0.8)
1.5 T / 3 T image pairs	35 / 31	29 / 27	27 / 11	22 / 20	**4 / 14**	2 / 4	5 / 9	3 / 6	5 / 3	9 / 8	20 / 12
Scan acquisition dates	1992–2018	1992–2017	1993–2018	1995–2017	2005–2018	2005–2015	1992–2014	1996–2015	1995–2017	1992–2015	1993–2017

* Two rarer genetic causes of FTD (*TBK1* and *SQSTM1* mutations) with known TDP-43 pathology also included

AD – Alzheimer’s disease pathology

Measures in bold were significantly different between patients and controls and/or within patient subgroups based on baseline comparisons (see Results).

### 2.2 Image analysis

#### 2.2.1 Image acquisition

MR images were acquired using several 1.5 Tesla and 3 Tesla scanners (Supplementary Table S8). The majority of 1.5 T images were acquired on a GE Signa scanner (General Electric Medical Systems, Milwaukee, Wisconsin, USA) employing a spoiled gradient-echo technique. The 3 T images were acquired on either a 3 T Siemens Trio or Prisma scanner (Siemens, Erlangen, Germany) both employing an MPRAGE sequence. Repeat images were acquired on the same scanner as the baseline.

#### 2.2.2 Whole-brain segmentation

**Fig. 1:**
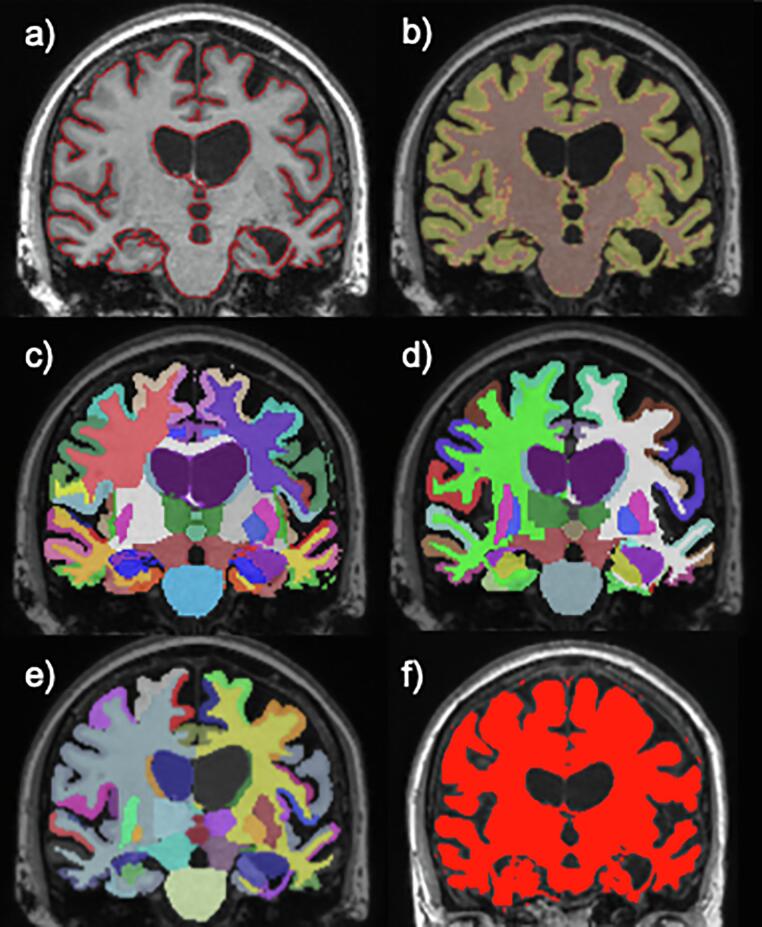
Representation of the image label outputs for the six automated whole-brain segmentation techniques on a randomly selected participant from the final cohort: a) BMAPS - Brain multi-atlas propagation and segmentations, b) SPM12 – Statistical Parametric Mapping Segment, c) GIF – Geodesic Information Flow, d) FS - Freesurfer, e) MALP-EM - Multi-Atlas Label Propagation with Expectation-Maximisation-based refinement f) SIENAX - Structural Image Evaluation, using Normalisation, of Atrophy- cross-sectional (MNI-152 space).

Six segmentation packages that are used widely in clinical research or employed in other neurodegenerative treatment trials were selected for comparison. These were: BMAPS (Brain Multi-Atlas Propagation and Segmentation (Leung et al., 2011)), SPM12 Segment (Ashburner and Friston, 2005) using MATLAB 14.1, GIF (Geodesic Information Flows) parcellation v3 (Cardoso et al., 2015), Freesurfer v5.3 (Fischl et al., 2002, Fischl et al., 2004), MALP-EM (Multi-Atlas Label Propagation with Expectation-Maximisation-based refinement) v2.1 (Ledig et al., 2015), and SIENAX (Structural Image Evaluation, using Normalisation, of Atrophy – cross-sectional (Smith et al., 2002, 2007)) (Fig. 1)

All images underwent an N4 bias correction for inhomogeneity (Tustison et al., 2010) and the segmentation pipeline applied independently to all 524 images (262 baseline and repeat pairs). BMAPS, Freesurfer, GIF, MALP-EM and SPM12 Segment were all applied to the native space images using default or developer recommended pipelines and options. The SIENAX pipeline was applied to images pre-aligned to MNI-152 template space to ensure the FLIRT and BET stages of the pipeline completed successfully. This pre-registration to MNI template space was required as a final trouble-shooting solution to the SIENAX pipeline failing to complete in native space across the different scanner pairs uniformly on this multi-scanner cohort. A representation of the output for the six segmentation methods can be seen in Fig. 1. To obtain a single whole brain region, some methods were summed together, either by tissue type (GM + WM) or by merging all appropriate brain region labels together.

#### 2.2.3 Determining longitudinal rates of atrophy

The resulting segmentations from these six automated techniques were used to derive seventeen indirect and direct measures of longitudinal volume change as summarised in Table 2.

**Table 2:** Methods used to derive indirect and direct measures of longitudinal change in whole brain volume for each segmentation technique.

Segmentation technique	Indirect brain volume (BV) change measure	Direct atrophy measure		
		K-means Boundary Shift Integral (KBSI)	Generalised Boundary Shift Integral (GBSI)	Integrated PBVC longitudinal pipeline
BMAPS - Brain Multi-Atlas Propagation and Segmentations	✓	✓	✕	✕
	BMPS_BV	BMAPS_KBSI		
FS - Freesurfer (v5.3)	✓	✓	✕	✕
	FS_BV	FS_KBSI		
GIF - Geodesic Information Flow	✓	✓	✓	✕
	GIF_BV	GIF_KBSI	GIF_GBSI	
MALP-EM - Multi-Atlas Label Propagation with Expectation-Maximisation-based refinement	✓	✓	✓	✕
	MALP-EM_BV	MALP-EM_KBSI	MALP-EM_GBSI	
SIENAX - Structural Image Evaluation, using Normalisation, of Atrophy- cross-sectional	✓	✓	✕	✓
	SIENAX_BV	SIENAX_KBSI		SIENA_PBVC
SPM - Statistical Parametric Mapping	✓	✓	✓	✓
	SPM_BV	SPM_KBSI	SPM_GBSI	SPM_PBVC

For the six indirect brain volume difference measures (BMAPS_BV, Freesurfer_BV, GIF_BV, MALP-EM_BV, SIENAX_BV, SPM_BV), the repeat volume was subtracted from the baseline volume for the image pair, using each of the six segmentation techniques and expressed as an annual percentage change from baseline.

(1) $$\mathrm{Indirectannualised}\%change=\frac{\left(v_{1}-v_{2}\right)/v_{1}}{\Delta t}*100$$

Where,

$v_{1}$ – volume at baseline

$v_{2}$ – volume at repeat scan

Δt – time interval between scans (years)

Nine direct measures of change were investigated using the boundary shift integral (BSI) (Freeborough and Fox, 1997, Leung et al., 2010, Prados et al., 2015), which measures the intensity profile shift at the brain boundary between two spatially aligned images and directly quantifies the equivalent volume change at this structural boundary. For the BSI pipeline, each image and segmentation pair underwent a symmetric affine rigid 12 dof (degrees-of-freedom) registration to spatially align the baseline and follow-up inputs. The transformation parameters for this registration were averaged to ascertain the midpoint space. This has been shown to avoid bias compared to uni-directional (non-symmetric) transformation (i.e follow-up to baseline alignment). Once transformed into the midpoint space, both images underwent differential bias correction to further match intensity profiles of tissue types across and between the images. Volume change was measured in the average space between the image pairs (Leung et al., 2012). A strength of the BSI pipeline is that it can be applied using a variety of regional binary or probabilistic mask as the initial ROI (region of interest) used to inform the area over which the boundary shift is quantified. The whole-brain segmentations derived from the six segmentation methods were each used separately as this initial ROI input for the pipeline. To help account for partial volume and segmentation errors, the K-means or KBSI (Leung et al., 2010) performs a tissue-specific intensity normalization and parameter selection for quantifying atrophy at the boundary informed using the binary segmentation masks. A recent development on this work is the generalised or GBSI, which adaptively estimates a non-binary ROI from probabilistic brain segmentations to allow for greater flexibility and accuracy in localizing and capturing atrophy (Prados et al., 2015).

All six techniques produced a binary mask, which was used to calculate the KBSI. SPM, GIF and MALP-EM also produced probabilistic masks used for the additional three GBSI measures (Table 2). The BSI pipelines produce ml change in brain volume, which was then converted into percentage of baseline volume change:

(2) $$\mathrm{BSIannualised}\%change=\frac{\left(\Delta v\right)/v_{1}}{\Delta t}*100$$

Where,

Δv – BSI derived change in brain volume (ml)

The longitudinal pipelines for SIENA and SPM were also applied to derive the final two direct measures of volume change. The longitudinal SPM pipeline produces an annualised percentage brain volume change (Long_SPM_PBVC), whilst the percentage brain volume change calculated by SIENA was annualised using the scan interval for each image pair (SIENA_PBVC):

(3) $$\mathrm{SIENAannualised}\%change=\frac{\Delta pc}{\Delta t}*100$$

Where,

Δpc – SIENA derived change in brain volume (%)

To ensure the integrity of the imaging data, all automated brain segmentations, KBSI and GBSI registrations and longitudinal SIENA and SPM pipeline outputs were visually assessed for errors or pipeline failures by an experienced image analyst (EG), blinded to patient and diagnosis. The longitudinal BSI registration review involved loading both baseline and co-registered repeat image, with the accompanying paired segmentation (BMAPS, Freesurfer, GIF, MALP-EM, SIENAX or SPM) into a 3D viewer that enabled switching between both images of the co-registered pair to assess any non-biological geometric distortion between timepoints that would result in an unreliable BSI. If an image pair failed (either due to cross-sectional or longitudinal quality control (QC) or for pipeline performance failures: see Supplementary Table S7), the participant was dropped from the final analysis. A common cohort of 226 participants, each with a full set of 17 passing automated longitudinal measures of brain atrophy, were included in the final analysis cohort (Table 1).

#### 2.2.4 Statistical analysis for comparing neuroimaging outcome measures

All statistical analyses were performed using STATA version 14.1 (Stata Statistical Software: College Station, TX: StataCorp LP). Baseline demographic variables were compared using a Kruskal-Wallis test for the continuous variables and a Fisher’s exact test for the categorical variables of sex and scanner type. Linear regression was used to compare the mean annualised rate of atrophy, adjusting for age, sex and scanner type. Controls were compared with i) the *clinical* subgroups, ii) the *genetic* subgroups and iii) the *pathologically-confirmed* subgroups in three separate tests. Comparisons of the patient subgroups *within* each of these three groups was performed (i.e. comparing the three genetic subgroups), but comparisons of the subgroups *across* the clinical, genetic and pathology groups was not.

Sample sizes were calculated to detect a 30% reduction in disease progression (as measured using annualised atrophy rate), corrected for the corresponding control rate of volume change for each whole-brain atrophy measure. These calculations assume a 1:1 randomisation into control and active treatment groups. This was performed as follows:

Initially, the effect size was calculated using the mean difference between patients and controls,${\mu_{p}-\mu }_{c}$ and the standard deviation of the patient subgroup,σ for each of the 17 whole-brain atrophy rates:

(4) $$\mathrm{ES}=\frac{{\mu_{p}-\mu }_{c}}{\sigma_{p}}$$

Where,

ES – Effect size

$\mu_{p}$ – Mean annualised % change in brain volume in patients

$\mu_{c}$ – Mean annualised % change in brain volume in controls

$\sigma_{p}$ – standard deviation of % change in brain volume in patients

The effect size was then converted into estimates for the sample size per treatment arm for a trial with equal allocation ratio to have 80% power to detect a 30% reduction in the annualised atrophy rate for treatment versus control at the conventional 5% significance level:

(5) $$n=\frac{2\sigma^{2}}{{\left(0.30\left({\mu_{1}-\mu }_{0}\right)\right)}^{2}}*7.85=\frac{2}{(0.30E{S)}^{2}}*7.85$$

To perform a head-to-head comparison between methods, the ratio between the sample sizes was used. All combinations between each of the seventeen different methods was computed, resulting in a total of (17*16)/2 = 136 head-to-head comparisons. These comparisons were performed separately for the clinical, genetic and pathology subgroups.

Bias-corrected and accelerated (BCa) bootstrapping was used to provide 95% confidence intervals for the sample size and ratios of sample sizes, with 2000 replications, stratified by diagnosis, genetic status and pathology patient subgroups (Carpenter and Bithell, 2000). To provide a better approximation to a normal distribution, the bootstrap confidence intervals were calculated for the effect size instead of sample size and for the natural logarithm of the ratio of effect sizes instead of ratio of sample sizes. The latter was chosen because it has a direct relationship with the ratio of sample sizes for the two methods being compared:

(6) $$\frac{n_{1}}{n_{2}}=\frac{2/{\left(0.30{ES}_{1}\right)}^{2}*7.85}{2/{\left(0.30{ES}_{2}\right)}^{2}*7.85}=\frac{1/{ES}_{1}^{2}}{1/{ES}_{2}^{2}}={\left(\frac{ES_{2}}{ES_{1}}\right)}^{2}$$

The upper and lower limits for the confidence intervals for the sample size and ratios of sample sizes were calculated by back transforming the upper and lower limits of the confidence interval for the effect size and natural log of the ratio of effect sizes, respectively.

## 3 Results

### 3.1 Participants

There were several significant differences in baseline demographics between healthy controls and FTD subgroups, as well as between the patient subgroups (bold in Table 1). In particular, there were differences in sex distribution, age at baseline, and age at symptom onset. Disease duration at baseline differed between the clinical and genetic patient subgroups but not between pathology subgroups. Finally, the lvPPA subgroup had a greater proportion of scans using 3 T vs 1.5 T image acquisition, compared to both controls and the other clinical FTD subgroups. LvPPA was described in 2004 (Gorno-Tempini et al., 2004, Gorno-Tempini et al., 2011) resulting in higher proportion of 3 T acquisitions. To account for these between group differences, age, sex and scanner type were included as covariates in all regression analyses.

### 3.2 Differences in rates of whole-brain atrophy

**Fig. 2:**
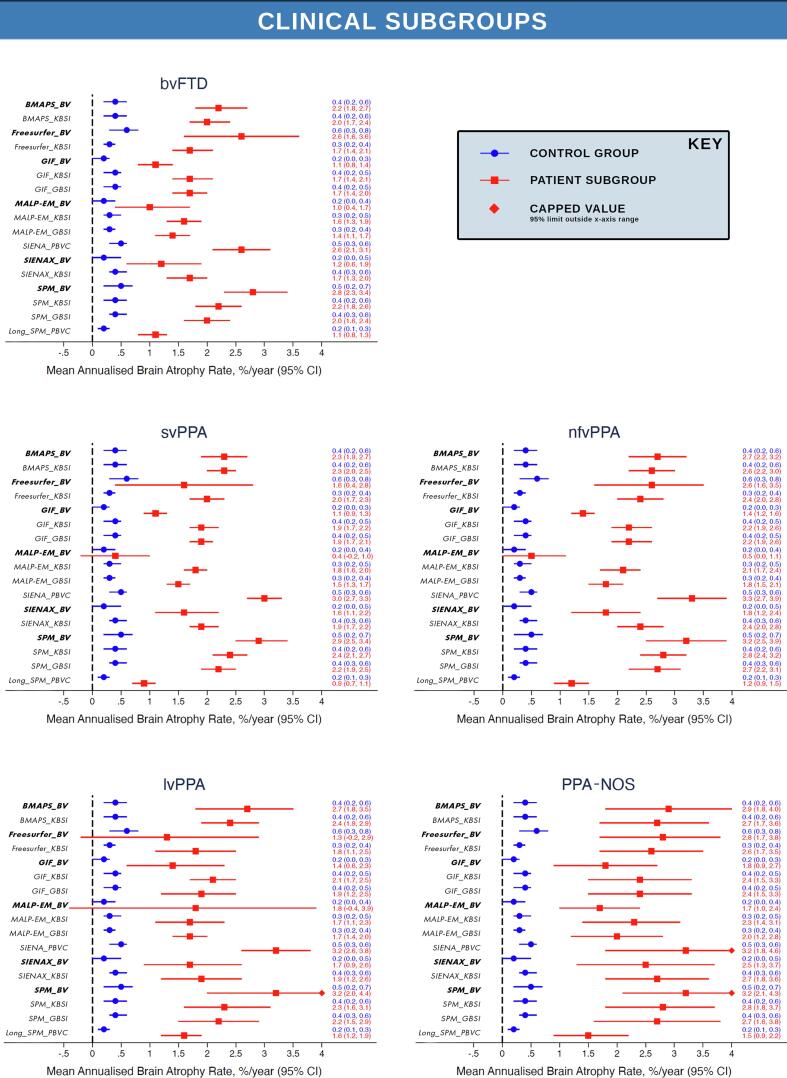

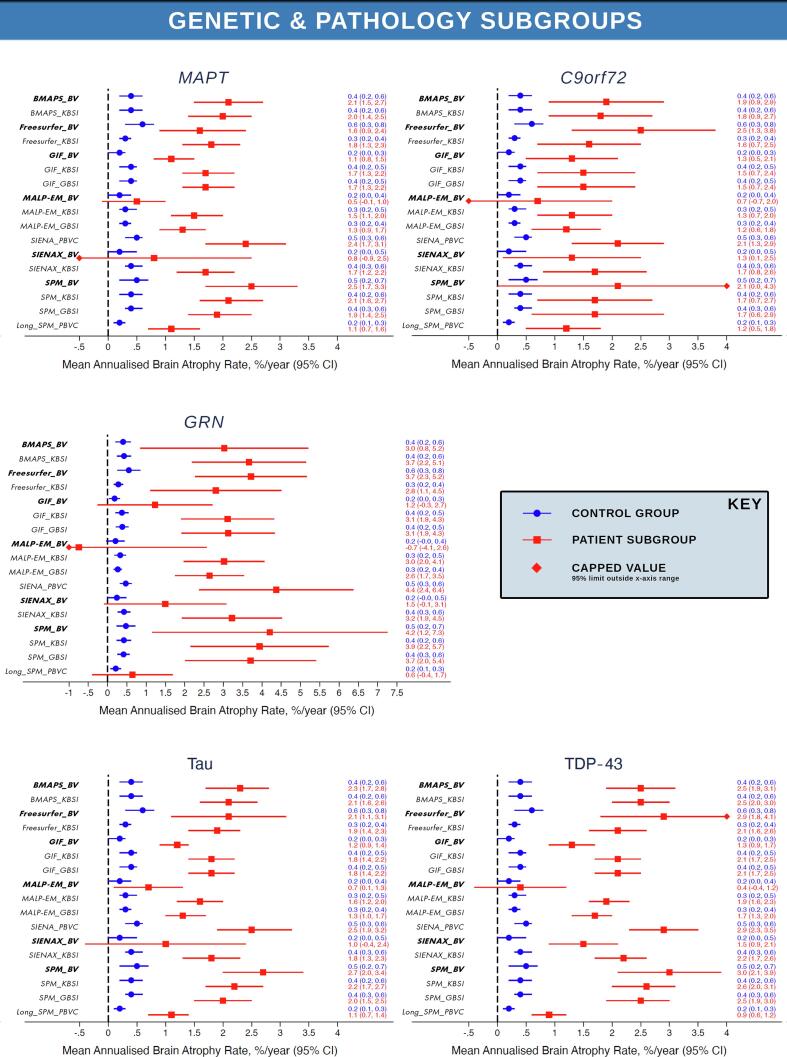
Mean and 95% confidence interval for annual whole-brain atrophy rate for all longitudinal measures for controls (blue) and each FTD subgroup (*Indirect _BV measures are bold and italiced to aid visual review). Mean and 95% confidence interval for annual whole-brain atrophy rate for all longitudinal measures for controls (blue) and each FTD subgroup. **Note the extended x-axis for the *GRN* subgroup to accommodate the substantially higher annual atrophy rates and wider BCa confidence intervals. (For interpretation of the references to colour in this figure legend, the reader is referred to the web version of this article.)

Fig. 2 presents the mean rates of whole-brain volume change with 95% confidence intervals for controls and patients across all 17 longitudinal measures separately for each FTD subgroup. These results demonstrate there was good group separation of controls and patients demonstrated for all longitudinal measures, with the exception of the indirect measures MALP-EM_BV, Freesurfer_BV and SIENAX_BV for the majority of clinical, genetic and pathological subgroups. Control rates of change (blue in Fig. 2) were relatively homogeneous across the measures with consistently low mean annualised rate of change close to 0. The highest standard deviations relative to mean change were for MALP-EM_BV and Freesurfer_BV measures. As indirect volume difference measures, this increased noise was due to the higher variability in the cross-sectional segmentation quality for these methods. Freesurfer had some of the highest mean-to-sd ratios in the raw baseline and repeat segmentation volumes, with MALP-EM segmentations consistently overestimating the brain boundary to include the largest amount of dura, as demonstrated by the substantially higher whole-brain volumes across all subgroups shown in Supplementary Table S3. Applying the direct BSI measures to both these segmentation methods substantially improved the mean-to-sd ratio, providing a more robust and consistent measure of longitudinal change. A full summary of the mean and sd atrophy rates for each of the 17 longitudinal measures across the 10 patient subgroups and controls are summarised in Supplementary S4.

In contrast to the controls, annual rates of change derived from the 17 methods showed more heterogeneity within and between each patient subgroup. In the clinical subgroups, mean rate of annual atrophy across all methods was the lowest overall mean rate of 1.8 (1.6)% for the bvFTD subgroup, and demonstrated the lowest mean-to-sd ratio. SvPPA mean rates were 1.9 (1.2)%, nfvPPA showed a mean rate of 2.2 (1.5)%, lvPPA showed a 2.1 (1.6)% mean annual change and the PPA-NOS subgroup showed the highest mean rate of 2.5 (0.9)%. When considering the range of values provided by each measure within a subgroup, MALP-EM_BV consistently provided the lowest raw mean annual rates of change, with either SPM_BV or SIENA_PBVC reporting the highest (Supplementary Table S4).

For the genetic subgroups, the mean annual rate derived across all methods was 1.5 (1.2)% for the *MAPT* subgroup, 1.7 (1.4)% for the *C9orf72* subgroup, and 3.0 (2.0)% for the *GRN* subgroup. Mean rates within the pathology subgroups in part reflected the contributions of the genetic subgroup results with the Tau subgroup presenting with a mean rate of 1.6 (1.2)%, similar to the *MAPT* results, whilst the TDP-43 subgroup reported a higher mean with 2.0 (1.6)%, reflecting the combined contribution of the *C9orf72* and *GRN* individuals along with the sporadic patients who had come to postmortem. Again, MALP-EM_BV universally provided the lowest annual rates of change and either SPM_BV or SIENA_PBVC consistently provided the highest mean rate of change.

There was generally good group separation of controls and patients for all longitudinal measures for the clinical, genetic and pathological FTD subgroups as presented in Fig. 2. Results were similar after adjusting for age, sex and scanner type in the linear regression analysis. These analyses demonstrated there were highly significant differences (*p* less than 0.001) between all clinical, genetic and pathology patient subgroups and controls for almost all longitudinal measures, with the exception of the indirect measure using MALP-EM_BV, SIENAX_BV and Freesurfer_BV (Supplementary Table S6 provides the full regression results).

### 3.3 Sample size results and comparison of techniques

Table 3 reports the sample size per arm required by each of the 17 atrophy measures to detect a 30% reduction in annualised atrophy rate for treatment versus control (with 80% statistical power at 5% significance level), after accounting for the rate of volume change in controls. The best performing longitudinal technique as determined by providing the smallest sample size point estimate is highlighted in blue in Table 3. This differed between the clinical, genetic and pathological subgroups but importantly, in all cases, the sample size required by this method was not significantly smaller than several other longitudinal measures (highlighted in green and underlined in Table 3), which each provided equivalent point estimates with overlapping 95% CI. Indirect methods generally had larger sample size estimates than their direct counterparts, suggesting application of the BSI or longitudinal pipelines produced a substantially more robust atrophy measure. Of the indirect measures, BMAPS, GIF and SPM segmentations performed better as longitudinal measures of volume change than Freesurfer, SIENA and MALP-EM segmentations. Direct KBSI, GBSI and PBVC measures were generally not significantly different to each other within each subgroup based on the paired comparisons (*p* > 0.05), although the longitudinal SPM pipeline (Long_SPM_PBVC) performed poorly for some of the patient subgroups.

**Table 3:** Sample size per arm to detect a 30% treatment effect, with 80% statistical power at 5% significance level, with 95% BCa CIs. Results highlighted in **blue and bold** produced the lowest sample size for each patient subgroup. Those in green and underlined did not significantly differ from the lowest estimate. Those in black produced significantly higher sample sizes than these lowest values but were significantly lower than those highlighted in red and italics, which produced the highest estimates or where the upper limit of the 95% CI included infinite sample size because there was not a significant difference between patient and control groups in the atrophy rate.

Sample size estimates were low and feasible across all patient subgroups, with the PPA-NOS subgroup providing the smallest estimate at 25 [95% CI (11–44)] patients required per treatment arm using the GBSI applied to SPM segmentations (SPM_GBSI). All 17 measures provided consistently low estimates for this patient subgroup. The more heterogenous bvFTD subgroup needed larger samples sizes to detect an equivalent treatment effect. The best performing technique (BMAPS_KBSI) in the bvFTD subgroup would require 114 individuals per treatment arm, whereas all other FTD patient groups required less than half this number using the best performing longitudinal measure. Sample size estimates for the lvPPA subgroup were generally higher than the other PPA subgroups, although still low for the best measure at 35 [22 – 68] participants per treatment arm using the GIF_GBSI. In the genetic groups, the longitudinal measures generally produced larger sample sizes for the *C9orf72* subgroup and in the TDP-43 pathology subgroups, with MALP-EM_BV producing the largest sample size estimate of all the measures across all the subgroups.

Overall, the methods providing the highest sample size point estimates, or where the 95% confidence for sample size included infinity (due to effect size confidence interval including zero – see Supplementary Table S5), were the indirect measures, particularly using MALP-EM, SIENAX and Freesurfer segmentations. In addition, the direct Long_SPM_PBVC measure performed poorly in the overlapping *GRN* and TDP-43 subgroups. The overall mean rates of change were higher in the SIENA_PBVC compared with the other direct longitudinal measures. Visual assessment of all 226 longitudinal SIENA_PBVC outputs did not indicate any clear registration inaccuracies that would preclude them from inclusion or suggest the results were an unreliable overestimation for any pair. The direct head-to-head comparisons with SIENA_PBVC demonstrated that despite these higher rates, the effect and sample sizes did not significantly differ from the KBSI, GBSI or SPM_PBVC results for many of the patient subgroups, resulting in a similar overall performance in terms of differentiating controls and patients and resulting sample size estimates. The BSI measures provided the lowest estimates or were not significantly different from the lowest estimates, and the indirect measures for BMAPS, SPM and GIF segmentations provided significantly lower estimates than indirect measures derived from the other segmentation methods across the patient subgroups.

## 4 Discussion

In this study we found that six fully automated segmentation techniques, which are widely used in clinical research, performed well at delineating the brain from non-brain for each of the images (particularly using BMAPS, GIF and SPM techniques). Despite good segmentation quality cross-sectionally, there were considerable differences in longitudinal performance of these techniques across the clinical, genetic and pathology FTD subgroups. Application of direct measures, such as the BSI, significantly reduced noise compared to indirectly measured volume change, as demonstrated by substantial increases in the mean-to-sd ratio and reduced sample size requirements for clinical trials.

During cross-sectional QC, only four segmentations (all using the Freesurfer pipeline) failed due to considerable exclusion of temporal lobe tissue. This segmentation issue has previously been reported for Freesurfer in a large Huntington’s Disease (HD) study (Johnson et al., 2017) and is a known issue in FTD given the often extensive focal temporal lobe atrophy evident on patient MR images. Previous cross-sectional comparative studies of automated segmentation techniques are relatively limited but have also shown that whilst techniques perform well at delineating structural images, there are subtle but important differences in accuracy and reliability. For example, Fellhauer and colleagues (Fellhauer et al., 2015) found that when applied to people with Alzheimer’s disease (AD) and mild cognitive impairment as well as healthy controls, Freesurfer produced the largest GM volumes alongside the smallest WM volumes and SPM produced the largest WM volumes. Scan quality was also an issue, with SPM providing the most accurate segmentations when image quality was poor, which is consistent with the current study’s results for the bvFTD subgroup whose images had the most significant motion artefacts compared with the PPA groups and where SPM performed comparatively well. Cross-sectional comparative studies have also shown significant differences in segmentation accuracy and reliability depending on the software version, operating system and workstation type (Gronenschild et al., 2012); as well as highlighting the importance of visual inspection of automated pipelines (Iscan et al., 2015). Both of these issues were addressed in the current study by applying all techniques in the same working environment and extensive visual assessment to provide as unbiased a comparison across measures and patient subgroups as possible. Whilst differences in GM/dura inclusion and minor segmentation errors were noted across all techniques and groups, manual editing was not performed to avoid any bias to the subset of images that would be chosen for such correction. In addition, the aim was to assess fully automated pipelines with the view that they could be applied by any trial site irrespective of whether an experienced image segmenter was available.

Despite good segmentation quality cross-sectionally, the level of delineation accuracy becomes much clearer when investigating longitudinal performance across both images in the scan pair. The variability in the indirect measures, which are derived solely from the segmentation volumes and do not include additional ‘direct’ information from the images themselves, demonstrates that even the slightest errors of additional dura or excluded brain matter can have a substantial impact on detection of underlying volumetric changes longitudinally. MALP-EM_BV consistently produced the lowest rate of annual volumetric change (Supplementary Tables S3 and S4). This was driven by both baseline and repeat images overestimating the GM boundary to include more dura than the other techniques, resulting in the largest and least accurate volumes across the subgroups. This issue has previously been reported in a population of HD patients and controls (Johnson et al., 2017). The application of the BSI, even to the segmentations that were less accurate, significantly reduced noise as demonstrated by substantial increases in the mean-to-sd ratio of atrophy measured. This produced larger effect sizes and considerably reduced sample size estimates to detect a treatment effect, with clear narrowing of the 95% BCa confidence intervals for these estimates. Thus, ultimately the BSI requires a reasonable, but not necessarily highly precise segmentation, to accurately inform an estimate of where atrophy is occurring and produce a robust measure of anatomical change between scans. This is also clear when comparing the longitudinal SIENA pipeline results with those produced using the indirect SIENAX_BV measure, which had a relatively poor performance across the subgroups. The developers have previously reported a strong correspondence with SIENA and the BSI when measuring whole-brain volume change (Smith et al., 2007). This remained evident based on the current results, with the SIENA longitudinal pipeline performing at least as well as many of the KBSI and GBSI atrophy measures, irrespective of initial segmentation used to inform the BSI algorithm.

The number and scope of people with FTD included in the current cohort and the ability to perform a direct head-to-head comparison of techniques on an identical dataset is a key strength of this study. This aimed to address the issue that, to date, considerable variability and inconsistency of rates of change results in FTD have likely arisen from investigations using small cohort numbers and application of techniques that could not be directly compared across studies. Reported rates of annualised volume loss in FTD substantially vary across studies (Supplementary Table S1). However, there is broad agreement that the PPA groups generally demonstrate faster and more homogeneous rates of global change than the greater heterogeneity evident in bvFTD cohorts, generally in the range of 2–3% annual volume loss (Chan et al., 2001, Gordon et al., 2010, Rohrer et al., 2008, Whitwell et al., 2015a). Annual rates of change reported in the current study using the best performing measure for each clinical subgroup also fall within this observed range, demonstrating greater group separation in the language variants from controls and lower sample sizes required to detect a putative treatment effect. The current study provides one of the first reports of global rates of atrophy in PPA-NOS patients, which demonstrated high and consistent volume loss using all seventeen atrophy measures (mean 2.5 (0.9)%). Two PPA-NOS patients had a *GRN* mutation, which is known to demonstrate particularly high rates of atrophy, and it is important to highlight that these initial results are based on a small cohort (n = 6). This caveat is particularly important when considering the resulting effect and sample size calculations derived from these rates of change. Given the small cohort size, bootstrap 95% CI’s may have had worse performance (e.g. lower coverage) than for the larger subgroup cohorts. These PPA-NOS results will need further validation in a larger cohort and caution is advised in interpretating these results given the current cohort size.

Previous longitudinal investigations involving patients with a known mutation are limited but show a similar range of global volumetric changes, suggesting *MAPT* mutations are associated with a mean annual rate of ∼ 1.6%, intermediate between those with *GRN* mutations, who exhibit the fastest rate of loss at ∼ 3.5% and *C9orf72* whose volumetric rates have been reported as the lowest at ∼ 1.4% and more in line with people with AD (Gordon et al., 2010, Rohrer et al., 2010a, Whitwell et al., 2015a, Whitwell et al., 2011). Using an average of the best performing direct measure, the current study reports a similar pattern of results, with annual atrophy rates of 1.8 (0.9)% in *MAPT*, 1.6 (1.1)% for *C9orf72* and 3.1 (2.0)% for *GRN* patients, who conclusively exhibit the highest rate of change across subgroups. With currently limited longitudinal data in FTD, it is reassuring these results are consistent with previous reports and provide additional valuable data in terms of variability in measurement and confidence intervals to better inform trial design and build on the current knowledge of disease progression across the FTD spectrum.

Direct comparison of automated longitudinal atrophy measures has not previously been investigated in FTD and showed significant differences in the performance across the 10 FTD subgroups in the current study. Initial positive therapeutic effects may translate to small changes in the rate of atrophy. Given the variability of performance, the importance of informed neuroimaging biomarker choice will be crucial to improve the chance of detecting any such disease-modifying effect. For a trial enrolling any of the patient subgroups, application of the BSI (GBSI where possible) to BMAPS, GIF or SPM segmentations overall produced the fewest subjects needed to detect a treatment effect with subtle but noteworthy differences between groups (Table 3). Reassuringly, estimates for the technique that performed best in each subgroup were low, ranging from 25 [11–44] for PPA-NOS to 114 [74–198] for the bvFTD group. In the genetic analysis, the *GRN* subgroup produced the lowest sample size with 35 [17–98] patients per treatment with the *C9orf72* subgroup providing the highest but still feasible estimate of 77 [25–378] patients required for enrolment per arm using the best method. Overall, the current study confirms the potential value of fully automated whole-brain atrophy measures as potential biomarkers across the FTD spectrum. Given FTD is characterised by often focal patterns of volume loss, similar comparative studies assessing the value of more regional and subcortical measures of change as potential outcomes across the subgroups may prove similarly informative for the design of future sporadic and genetic FTD trials.

The current study reported sample sizes that were not dissimilar to previous studies enrolling larger patient cohorts and employing the BSI method. However, compared with studies investigating smaller cohorts, the current sample sizes are considerably lower across many of the clinical subgroups for the best performing method. To date, only one study has reported global rates of change in the different genetic populations with accompanying sample size estimates (Whitwell et al., 2015a), applying the BSI to SPM segmentations to derive the results. The current study produced substantially lower estimates for the *MAPT* subgroup and equivalent sample sizes for the *C9orf72* and *GRN* subgroups using this direct measure of change. Despite equivalent participant numbers, there were clear differences between the age of participants, disease duration and scan interval compared with the current study making a meaningful direct comparison difficult. However, the key results of both these previous estimates and the current study demonstrate the utility of direct automated longitudinal measures of whole-brain volume change as potential non-invasive markers for upcoming genetic FTD trials.

A key aim of this study was to assess the utility of these fully automated measures as potential biomarkers and this performance was evaluated based on sample size calculations. However, when evaluating biomarker choice, there are other practical factors such as ease of application to consider. This will be particularly relevant in multicentre trials of a rare disease such as FTD, where some recruitment sites may not have experienced image analysts to run these techniques and troubleshoot when pipelines fail to complete. One SPM segmentation failed to complete and additional options were required to obtain the full-brain mask in MALP-EM. However, the SIENAX segmentation pipeline required considerable troubleshooting for successful completion uniformly across all image pairs with the same pipeline settings, with the final solution being a pre-alignment of all images into MNI-152 space prior to application of the pipeline. Given this consideration of needing to apply these techniques reliably, easily and repeatedly across multiple sites, scanners, and acquisitions regardless of available expertise at the scanner site, the current study suggests that SPM, GIF and BMAPS stand out as preferable automated segmentation techniques based on this dataset.

Another important issue highlighted by the current results relates to reporting and interpreting of sample sizes more generally. Issues with the reporting of sample sizes have been previously raised in the literature, demonstrating that many articles in high-impact medical journals failed to provide adequate or accurate reporting (Charles et al., 2009). The current dataset has a number of examples of sample size estimates where the point estimate appears feasible but the realistic utility of these methods as evidenced by the potential upper 95% BCa CI limit suggests differently. In the lvPPA subgroup the GIF_KBSI measure provides a relatively low point estimate of 126 participants required per treatment arm. Based purely on the point estimate and the results demonstrating that the GIF_KBSI generally provides a robust measure of change, it appears this would be a reasonable biomarker choice for an upcoming trial enrolling these patients. However, the upper 95% BCa CI shows as many as 7760 participants may potentially be required to detect the treatment effect, demonstrating how misleading reliance on the estimate alone can be, particularly when sample sizes are based on limited data. In a genetic FTD trial enrolling participants with *MAPT* mutations, it appears both SIENA_PBVC and SPM_KBSI would be equivalent biomarker choices with a point estimate of 69 and 70 respectively. Although not as problematic as the lvPPA example, the BCa CI for SIENA_PBVC is 30–368 compared with 37–155 for SPM_KBSI, demonstrating the possibility that over twice as many participants may need to be recruited to power a trial using the former outcome measure. None of the publications summarised in the Supplementary Table S1 provided confidence intervals for their sample size estimates. This lack of reporting confidence intervals makes interpretation and comparison of previously published results problematic, particularly if calculations were based on smaller cohorts as is common in rare conditions such as FTD. The current data highlights the importance of including confidence intervals for sample size calculations as standard given that they provide much more realistic data to inform the design of trials, biomarker choice and to accurately plan recruitment. This will be essential to avoid the costly and ethical issues related to underpowered trials.

Another important issue in clinical trials is the attrition rate. Here, sample sizes are corrected for control rates of change but do not include a putative attrition rate because it has yet to be established what the retention rate in a large FTD trial might be. This, of course, would depend on the severity of patients included, the treatment type, likely adverse effects, and the number of visits required (Grill et al., 2015, Grill and Karlawish, 2010), so the current results are likely to underestimate the final recruitment numbers required once accounting for patient drop-out. An important caveat to this study is that the images underwent QC before inclusion into the cohort. This curation naturally biases the sample size estimates to be lower given a trial would recruit individuals who may not provide a usable scan that is adequate for analysis. In addition, many of the participants were enrolled in ongoing longitudinal studies, resulting in 46 of the participants having multiple images as candidates for study inclusion. Whilst the authors attempted to limit any additional bias by including the earliest passing scans that did not differ considerably in quality, this additional refinement would not necessarily occur in a trial situation. Again, there are no published data on the prevalence of this pass rate in FTD; however, UCL has conducted extensive neuroimaging in its Longitudinal Investigation of FTD (LIFTD) observational study, where approximately 16% of scans failed QC at baseline before analysis (based on almost 300 patients enrolled, unpublished data). Importantly, most patients returned to repeat this imaging assessment and acquired a successful baseline scan, resulting in a low overall failure rate in this symptomatic FTD study. In fact, increasing in trials where imaging markers are primary outcomes or key endpoints for the analysis plan, there is an explicit requirement for a passing baseline scan prior to randomisation. For example in the MS-SMART trial ((Chataway et al., 2020) https://clinicaltrials.gov/ct2/show/NCT01910259), the volumetric imaging was acquired twice at baseline, with the best-quality scan chosen for analysis on the basis of expert visual rating of motion and artifacts that may preclude the delineation of the brain, in line with the QC conducted in the current study. In such trials, the current estimates would be fairly representative of those required to power a design with similar inclusion and exclusion criteria.

## 5 Conclusion

The results of this study confirm that fully automated methods for measuring longitudinal whole-brain atrophy perform well across all clinical, genetic and pathology subgroups in FTD. In the current study, whilst the best performing techniques vary across the different subgroups, there are multiple techniques for each that provide small and feasible sample sizes to detect a disease-modifying effect on global atrophy rate. The direct head-to-head comparison of techniques in this large FTD cohort demonstrates the importance of the choice of technique depending on the patient population being enrolled or investigated, which will be of value in informing biomarker choice in the future. In general, direct measures of change including application of the BSI to SPM, GIF or BMAPS segmentation outperformed the other direct and all indirect measures of change. Many of these segmentation and longitudinal pipelines worked ‘out of the box’, requiring little or no additional optimisation, making them ideal for application in multicentre FTD trials, which may include sites that do not have advanced image analysis expertise.

## CRediT authorship contribution statement

**Elizabeth Gordon:** Conceptualization, Methodology, Software, Validation, Formal analysis, Investigation, Data curation, Writing - original draft, Writing - review & editing, Visualization. **Martina Bocchetta:** Conceptualization, Methodology, Writing - review & editing, Supervision. **Jennifer Nicholas:** Software, Methodology, Writing - review & editing, Visualization, Supervision. **David M Cash:** Methodology, Writing - review & editing. **Jonathan D Rohrer:** Conceptualization, Investigation, Resources, Methodology, Writing - review & editing, Supervision.
