# Supplementary material for: A comparison of automated atrophy measures across the frontotemporal dementia spectrum: Implications for trials
Source: Neuroimage Clin. 2021 Oct 5;32:102842. doi: 10.1016/j.nicl.2021.102842 (PMC8503665; doi:10.1016/j.nicl.2021.102842)
Supplement: Supplementary data 1 [file mmc1.docx]

**Supplementary data**

**Table S1** Summary of previously published sample size estimates from studies investigating longitudinal global volumetric changes in FTD, split by patient subgroup. The power, β, level of treatment effect, statistical significance level, α, and additional corrections used for the calculation are listed under each publication. Updated and adapted from Gordon et al., (2016)

**Table S2** Retrospective FTD cohort demographic summary reporting mean (standard deviation) unless otherwise stated.

**Table S3** Mean and standard deviation (sd) for baseline (Bl) and repeat (Rp) segmentation volumes (mls) and annualised rates of change measures (expressed as a percentage of baseline volume change) for controls and FTD clinical, genetic and pathological subgroups (n = 262).

*** Obs =** number of passing observations used to produce results per technique demonstrating the distribution of measures per subgroup that failed, resulting in the exclusion of that participant from the final analysis n=226 cohort.

**Table S4** Mean and standard deviation (sd) of the annualised rate of change in whole-brain volume (%) for controls and the clinical, genetic and pathology FTD subgroups in the refined (n = 226) cohort.

**Table S5** Effect size calculations for the 17 atrophy measures used to derive the sample size results, with 95% BCa confidence intervals. Effect sizes greater than one are presented in bold as these are considered a large effect based on Cohen’s (1988) original formulation, suggesting good group separation between patients and controls

**Table S6** Linear regression analysis results reporting the difference in mean compared to controls [95% CI] for clinical, genetic and pathology FTD subgroups for all longitudinal annual whole-brain measures of change

**Table S7** Overall numbers of segmentations and registrations that failed for each of the six segmentation methods due to incomplete pipeline execution or QC issue.

**Table S8** Breakdown of scanner information slit by scanning period and displaying manufacturer, field strength and key acquisition parameters.

**Table S1** Summary of previously published sample size estimates investigating longitudinal global volumetric changes in FTD, split by patient subgroup. The power, β, level of treatment effect, statistical significance level, α, and additional corrections used for the calculation are listed under each publication. Updated from Gordon et al., (2016). Estimates are per treatment arm and assume an equal allocation ratio to active and placebo treatment groups.

| Publication, and cohort | Parameters | bvFTD | svPPA | nfvPPA | lvPPA | FTD-combined | *MAPT* | *C9orf72* | *GRN* |
| --- | --- | --- | --- | --- | --- | --- | --- | --- | --- |
| Rohrer et al. (2008) | β = 90%, α = 0.05, 30% treatment effect |  |  |  |  |  |  |  |  |
| svPPA (n = 21, 8 FTLD-U) |  |  | 118 |  |  |  |  |  |  |
| Knopman et al. (2009) | β = 80%, α = 0.05, 30% treatment effect*, correcting for 26% attrition rate |  |  |  |  |  |  |  |  |
| bvFTD (n = 34), svPPA (n = 16), nfvPPA (n = 17), lvPPA (n = 9) |  | 117 | 96 | 75 | 57 |  |  |  |  |
| Gordon et al. (2010) | β = 80%, α = 0.05, 30% treatment effect*, adjusting for control rates and annual attrition of 10% |  |  |  |  |  |  |  |  |
| bvFTD (n = 11), svPPA (n = 11), nfvPPA (n = 10) |  | 251 | 80 | 18 |  |  |  |  |  |
| Rohrer et al. (2012) | β = 90%, α = 0.05, 30% treatment effect, adjusting for control rates |  |  |  |  |  |  |  |  |
| svPPA (n = 17), nfvPPA (n = 18) 30% |  |  | 120 | 70 |  |  |  |  |  |
| Mahoney et al. (2015) | β = 80%, α = 0.05, 30% treatment effect, adjusting for control rates |  |  |  |  |  |  |  |  |
| bvFTD (n = 19, including 8 *MAPT* and 4 *C9orf72* mutations) |  | 226 |  |  |  |  |  |  |  |
| Whitwell et al. (2015) | β = 80%, α = 0.05, 30% treatment effect* |  |  |  |  |  |  |  |  |
| *MAPT* (n = 21), *C9orf72* (n = 11), *GRN* (n = 11), sporadic FTD (n = 15, 14 bvFTD and 1 FTD-ALS) |  |  |  |  |  | 39 | 106 | 60 | 45 |
| Pankov et al. (2016) | β = 80%, α = 0.05, 30% treatment effect* |  |  |  |  |  |  |  |  |
| bvFTD, n = 37 [14 in 1.5 T and 23 in 3 T]), svPPA, n = 49 [29 in 1.5 T and 20 in 3 T]) | 1.5 Tesla |  |  |  |  | 70 |  |  |  |
|  | 3 Tesla |  |  |  |  | 33 |  |  |  |

**Table S2** Full retrospective FTD cohort demographic summary (n=262) reporting mean (standard deviation) unless otherwise stated. Patients are split into groups based on their clinical diagnosis, genetic mutation status and those with known Tau or TDP-43 pathology (either pathological confirmation and/or genetic mutation status). Cells in bold were revealed to be significantly different at the p < 0.05 using the Kruskal-Wallis test for continuous variable and Fisher’s exact test for the categorical variables.

|  | **Healthy controls** | **Clinical FTD subgroups** | | | | | **Genetic FTD subgroups** | | | **Pathology FTD subgroups** | |
| --- | --- | --- | --- | --- | --- | --- | --- | --- | --- | --- | --- |
|  |  | **bvFTD** | **svPPA** | **nfvPPA** | **lvPPA** | **PPA-NOS** | ***MAPT*** | ***C9orf72*** | ***GRN*** | **Tau** | **TDP-43** |
| Number of participants | 78 | 66 | 45 | 45 | 21 | 7 | 16 | 10 | 8 | 19 | 34 |
| Clinical, genetic and pathology mapping | NA | 15 *MAPT*;  9 *C9orf72*;  4 *GRN*;  1 Tau;  2 TDP-43 Type A | 2 Tau;  10 TDP-43 Type C | 1 *C9orf72*;  2 *GRN*;  2 TDP-43 Type A | 5 AD;  1 *MAPT* | 2 *GRN* | 15 bvFTD;  1 lvPPA | 9 bvFTD;  1 nfvPPA | 4 bvFTD;  2 nfvPPA;  2 PPA-NOS | 16 *MAPT*;  3 post-mortem confirmed | 10 *C9orf72*;  8 *GRN*;  14 post-mortem confirmed * |
| Male/Female | 34 / 44 | **52 / 14** | 24 / 21 | 26 / 19 | **15 / 6** | 4 / 3 | 9 / 7 | **9 / 1** | 3 / 5 | 12 / 7 | 21 / 13 |
| % male | 44% | **75%** | 53% | 58% | **71%** | 57% | 56% | **90%** | 38% | 63% | 49% |
| Age at baseline | 62.3 (11.6) | **62.6 (8.6)** | 63.7 (7.8) | 65.6 (7.1) | 67.4 (7.4) | 62.6 (7.0) | **55.4 (7.2)** | 62.5 (6.0) | 61.2 (7.2) | **56.2 (7.2)** | 62.5 (6.4) |
| Age at symptom onset (years) | NA | 56.7 (8.8) | 59.0 (7.5) | 61.2 (6.8) | 63.3 (7.1) | 60.1 (7.5) | 49.1 (5.8) | 55.6 (8.6) | 58.0 (6.4) | 50.7 (7.0) | 57.7 (7.3) |
| Disease duration (years) | NA | **5.9 (3.9)** | 4.7 (1.9) | 4.4 (1.9) | 4.1 (1.9) | **2.4 (1.4)** | 6.3 (3.9) | 6.9 (4.4) | 3.2 (3.4) | 5.6 (3.7) | 4.8 (3.4) |
| Scan interval (years) | 1.5 (0.8) | 1.4 (0.8) | 1.5 (0.8) | 1.3 (0.6) | 1.3 (0.7) | 1.6 (0.7) | 1.9 (1.2) | 1.0 (0.4) | 1.2 (0.4) | 1.8 (1.2) | 1.5 (0.9) |
| 1.5T / 3.T image pairs | 40 / 38 | 34/ 32 | 29 / 16 | 23 / 22 | **4 / 17** | 2 / 5 | 6 / 10 | 4 / 6 | 5 / 3 | 10 / 9 | 21 / 13 |
| Scan acquisition dates | 1992 - 2018 | 1992 - 2017 | 1993 - 2018 | 1995 - 2017 | 2005 - 2018 | 2005 - 2015 | 1992 - 2014 | 1996 - 2015 | 1995 - 2017 | 1992 - 2015 | 1993 - 2017 |

* Two rarer genetic causes of FTD (*TBK1* and *SQSTM1* mutations) with known TDP-43 pathology also included

**Table S3** Mean and standard deviation (sd) for baseline (Bl) and repeat (Rp) segmentation volumes (mls) and annualised rates of change measures (expressed as a percentage of baseline volume change) for controls and FTD clinical, genetic and pathological subgroups (n = 262).

| **n=262** | **Controls (n=78)** | | | **Clinical subgroups** | | | | | | | | | | | | | | |
| --- | --- | --- | --- | --- | --- | --- | --- | --- | --- | --- | --- | --- | --- | --- | --- | --- | --- | --- |
|  |  |  |  | **bvFTD (n=66)** | | | **svPPA (n=45)** | | | **nfvPPA (n=45)** | | | **lvPPA (n=21)** | | | **PPA-NOS (n=7)** | | |
| **Measure** | **Obs** | **Mean** | **sd** | **Obs** | **Mean** | **sd** | **Obs** | **Mean** | **sd** | **Obs** | **Mean** | **sd** | **Obs** | **Mean** | **sd** | **Obs** | **Mean** | **sd** |
| **BMAPS_Bl** | 78 | 1115.7 | (103.0) | 66 | 1076.4 | (96.1) | 45 | 1088.7 | (98.2) | 45 | 1045.0 | (107.4) | 21 | 1059.5 | (100.1) | 7 | 1141.7 | (107.9) |
| **BMAPS_Rp** | 78 | 1108.2 | (102.5) | 66 | 1048.0 | (103.9) | 45 | 1050.4 | (100.2) | 45 | 1005.5 | (104.8) | 21 | 1026.2 | (103.0) | 7 | 1091.5 | (85.6) |
| **BMAPS_BV** | 78 | 0.4 | (0.9) | 66 | 2.0 | (1.8) | 45 | 2.4 | (1.2) | 45 | 2.9 | (1.8) | 21 | 2.5 | (1.6) | 7 | 2.8 | (1.0) |
| **BMAPS_KBSI** | 74 | 0.5 | (0.8) | 64 | 2.0 | (1.3) | 41 | 2.2 | (0.9) | 44 | 2.7 | (1.3) | 19 | 2.4 | (0.9) | 7 | 2.6 | (0.9) |
| **Freesurfer_Bl** | 77 | 1086.0 | (101.0) | 66 | 1067.7 | (101.5) | 45 | 1069.4 | (117.7) | 45 | 1042.9 | (120.4) | 21 | 1030.5 | (103.6) | 7 | 1115.7 | (114.0) |
| **Freesurfer_Rp** | 78 | 1075.8 | (99.1) | 64 | 1039.3 | (96.2) | 45 | 1037.3 | (127.8) | 45 | 1006.1 | (105.3) | 20 | 1007.4 | (97.1) | 7 | 1067.7 | (91.7) |
| **Freesurfer_BV** | 77 | 0.7 | (1.3) | 64 | 2.2 | (3.9) | 45 | 2.1 | (4.2) | 45 | 2.6 | (3.0) | 20 | 0.8 | (3.6) | 7 | 2.7 | (0.9) |
| **Freesurfer_KBSI** | 76 | 0.3 | (0.6) | 64 | 1.6 | (1.3) | 44 | 2.0 | (1.0) | 45 | 2.4 | (1.3) | 20 | 1.7 | (1.4) | 7 | 2.5 | (0.8) |
| **GIF_Bl** | 78 | 1115.5 | (98.9) | 66 | 1101.8 | (96.2) | 45 | 1107.3 | (108.5) | 45 | 1082.2 | (104.9) | 21 | 1103.0 | (109.3) | 7 | 1177.8 | (116.0) |
| **GIF_Rp** | 78 | 1112.2 | (98.5) | 66 | 1086.2 | (97.7) | 45 | 1089.1 | (107.3) | 45 | 1062.0 | (102.5) | 21 | 1084.6 | (113.9) | 7 | 1144.8 | (97.8) |
| **GIF_BV** | 78 | 0.2 | (0.6) | 66 | 1.1 | (1.2) | 45 | 1.1 | (0.7) | 45 | 1.4 | (0.7) | 21 | 1.2 | (1.7) | 7 | 1.7 | (0.8) |
| **GIF_KBSI** | 76 | 0.5 | (0.7) | 64 | 1.7 | (1.2) | 41 | 1.9 | (0.7) | 44 | 2.3 | (1.1) | 20 | 1.9 | (1.2) | 7 | 2.4 | (0.8) |
| **GIF_GBSI** | 73 | 0.4 | (0.7) | 63 | 1.7 | (1.2) | 43 | 1.9 | (0.7) | 44 | 2.3 | (1.1) | 20 | 2.0 | (0.8) | 7 | 2.4 | (0.8) |
| **MALP-EM_Bl** | 78 | 1135.7 | (107.6) | 66 | 1141.6 | (98.6) | 45 | 1140.1 | (107.6) | 45 | 1114.8 | (120.0) | 21 | 1119.0 | (112.8) | 7 | 1200.0 | (134.2) |
| **MALP-EM_Rp** | 78 | 1136.2 | (103.2) | 66 | 1126.2 | (95.5) | 45 | 1129.2 | (106.4) | 45 | 1104.2 | (114.2) | 21 | 1101.9 | (103.8) | 7 | 1172.1 | (120.1) |
| **MALP-EM_BV** | 78 | 0.0 | (1.8) | 66 | 1.1 | (2.5) | 45 | 0.5 | (1.9) | 45 | 0.6 | (1.8) | 21 | 1.3 | (4.3) | 7 | 1.6 | (0.7) |
| **MALP-EM_KBSI** | 75 | 0.3 | (0.5) | 63 | 1.4 | (1.0) | 45 | 1.5 | (0.6) | 42 | 1.8 | (1.0) | 20 | 1.7 | (0.6) | 7 | 2.0 | (0.7) |
| **MALP-EM_GBSI** | 72 | 0.4 | (0.7) | 62 | 1.6 | (1.1) | 45 | 1.8 | (0.7) | 44 | 2.1 | (1.1) | 19 | 1.7 | (1.1) | 7 | 2.3 | (0.7) |
| **SIENAX_Bl** | 78 | 1087.2 | (95.9) | 66 | 1069.3 | (91.6) | 45 | 1058.4 | (100.2) | 45 | 1037.1 | (106.9) | 21 | 1050.0 | (106.3) | 7 | 1124.6 | (107.9) |
| **SIENAX_Rp** | 78 | 1081.3 | (93.4) | 66 | 1055.9 | (100.3) | 45 | 1034.4 | (100.0) | 45 | 1011.3 | (99.1) | 21 | 1025.3 | (109.7) | 7 | 1083.3 | (88.4) |
| **SIENAX_BV** | 78 | 0.4 | (1.1) | 66 | 1.2 | (2.3) | 45 | 1.4 | (2.3) | 45 | 1.8 | (1.8) | 21 | 1.7 | (1.6) | 7 | 2.4 | (1.1) |
| **SIENAX_KBSI** | 77 | 0.5 | (0.7) | 65 | 1.6 | (1.2) | 42 | 1.9 | (0.8) | 44 | 2.5 | (1.2) | 21 | 1.9 | (1.4) | 7 | 2.6 | (0.8) |
| **SIENA_PBVC** | 78 | 0.5 | (0.7) | 66 | 2.4 | (1.9) | 45 | 3.0 | (1.0) | 45 | 3.3 | (2.0) | 21 | 3.0 | (1.3) | 7 | 3.2 | (1.2) |
| **SPM_Bl** | 78 | 1078.1 | (95.6) | 66 | 1019.7 | (98.8) | 45 | 1030.9 | (109.1) | 45 | 982.7 | (103.8) | 21 | 1003.8 | (99.2) | 7 | 1074.8 | (74.5) |
| **SPM_Rp** | 78 | 1069.1 | (95.0) | 66 | 984.5 | (113.2) | 44 | 986.4 | (114.2) | 45 | 938.9 | (109.3) | 21 | 960.2 | (107.4) | 7 | 1020.4 | (67.2) |
| **SPM_BV** | 78 | 0.6 | (1.0) | 66 | 2.8 | (2.4) | 44 | 3.1 | (1.8) | 45 | 3.2 | (2.3) | 21 | 3.6 | (2.7) | 7 | 3.3 | (1.0) |
| **SPM_KBSI** | 72 | 0.5 | (0.7) | 62 | 1.9 | (1.5) | 43 | 2.1 | (1.0) | 44 | 2.7 | (1.4) | 20 | 2.2 | (1.4) | 6 | 2.7 | (1.0) |
| **SPM_GBSI** | 71 | 0.5 | (0.8) | 63 | 2.2 | (1.5) | 43 | 2.4 | (0.9) | 44 | 2.9 | (1.4) | 20 | 2.4 | (1.5) | 7 | 2.8 | (0.8) |
| **Long_SPM_PBVC** | 78 | 0.2 | (0.6) | 66 | 1.0 | (0.9) | 45 | 1.0 | (0.7) | 45 | 1.2 | (1.0) | 21 | 1.5 | (0.7) | 7 | 1.6 | (0.6) |

*** Obs =** number of passing observations per technique demonstrating the distribution of measures per subgroup that failed and were thus excluded from the final analysis to produce the refined n=226 cohort.

**Table S3 continued** Mean and standard deviation (sd) for baseline (Bl) and repeat (Rp) segmentation volumes (mls) and annualised rates of change measures (expressed as a percentage of baseline volume change) for controls and FTD clinical, genetic and pathological subgroups (n = 262).

| **n=262** | **Genetic subgroups** | | | | | | | | | **Pathology subgroups** | | | | | |
| --- | --- | --- | --- | --- | --- | --- | --- | --- | --- | --- | --- | --- | --- | --- | --- |
|  | ***MAPT* (n=16)** | | | ***C9orf72* (n=10)** | | | ***GRN* (n=8)** | | | **Tau (n=19)** | | | **TDP43 (n=34)** | | |
| **Measure** | **Obs** | **Mean** | **sd** | **Obs** | **Mean** | **sd** | **Obs** | **Mean** | **sd** | **Obs** | **Mean** | **sd** | **Obs** | **Mean** | **sd** |
| **BMAPS_Bl** | 16 | 1052.3 | (87.8) | 10 | 1126.9 | (132.7) | 8 | 1040.7 | (118.1) | 19 | 1073.9 | (80.1) | 34 | 1099.4 | (123.9) |
| **BMAPS_Rp** | 16 | 1013.0 | (102.5) | 10 | 1103.4 | (141.2) | 8 | 997.9 | (112.6) | 19 | 1030.9 | (91.2) | 34 | 1059.2 | (128.2) |
| **BMAPS_BV** | 16 | 1.8 | (1.4) | 10 | 2.2 | (1.5) | 8 | 3.0 | (2.6) | 19 | 2.0 | (1.3) | 34 | 2.5 | (1.7) |
| **BMAPS_KBSI** | 16 | 1.8 | (1.0) | 10 | 2.0 | (1.3) | 8 | 3.7 | (1.8) | 19 | 1.9 | (1.0) | 33 | 2.5 | (1.4) |
| **Freesurfer_Bl** | 16 | 1034.0 | (84.9) | 10 | 1117.2 | (115.1) | 8 | 1031.1 | (112.7) | 19 | 1073.7 | (102.3) | 34 | 1091.8 | (127.9) |
| **Freesurfer_Rp** | 16 | 1007.7 | (96.1) | 10 | 1093.1 | (110.9) | 8 | 981.6 | (88.5) | 19 | 1037.2 | (89.3) | 34 | 1051.1 | (125.9) |
| **Freesurfer_BV** | 16 | 1.2 | (1.7) | 10 | 1.7 | (3.1) | 8 | 3.7 | (1.7) | 19 | 1.7 | (2.3) | 34 | 2.6 | (3.4) |
| **Freesurfer_KBSI** | 16 | 1.6 | (0.9) | 10 | 1.6 | (1.1) | 8 | 2.8 | (2.0) | 19 | 1.7 | (1.0) | 34 | 2.1 | (1.4) |
| **GIF_Bl** | 16 | 1069.3 | (76.5) | 10 | 1154.8 | (129) | 8 | 1072.4 | (130.2) | 19 | 1074.7 | (81.6) | 34 | 1123.6 | (129.7) |
| **GIF_Rp** | 16 | 1048.3 | (85) | 10 | 1139.7 | (135.6) | 8 | 1053.1 | (117.9) | 19 | 1052.9 | (84.4) | 34 | 1102.2 | (128.5) |
| **GIF_BV** | 16 | 1.0 | (0.6) | 10 | 1.4 | (1.0) | 8 | 1.2 | (1.8) | 19 | 1.1 | (0.6) | 34 | 1.3 | (1.1) |
| **GIF_KBSI** | 16 | 1.6 | (0.8) | 10 | 1.6 | (1.1) | 8 | 3.1 | (1.5) | 19 | 1.6 | (0.8) | 33 | 2.1 | (1.1) |
| **GIF_GBSI** | 15 | 1.7 | (0.8) | 10 | 1.6 | (1.1) | 8 | 3.1 | (1.4) | 18 | 1.7 | (0.8) | 34 | 2.1 | (1.1) |
| **MALP-EM_Bl** | 16 | 1098.7 | (80.6) | 10 | 1200.9 | (116.2) | 8 | 1089.7 | (164.9) | 19 | 1120.9 | (78.2) | 34 | 1155.4 | (139.1) |
| **MALP-EM_Rp** | 16 | 1086.2 | (92.5) | 10 | 1192.2 | (118.8) | 8 | 1089.6 | (134.4) | 19 | 1105.5 | (85.9) | 34 | 1144.4 | (128.6) |
| **MALP-EM_BV** | 16 | 0.4 | (1.0) | 10 | 0.7 | (1.7) | 8 | -0.7 | (4.0) | 19 | 0.6 | (1.1) | 34 | 0.4 | (2.2) |
| **MALP-EM_KBSI** | 16 | 1.2 | (0.7) | 10 | 1.2 | (0.7) | 8 | 2.6 | (1.1) | 19 | 1.2 | (0.7) | 33 | 1.7 | (0.9) |
| **MALP-EM_GBSI** | 16 | 1.4 | (0.9) | 10 | 1.4 | (0.9) | 8 | 3.0 | (1.3) | 19 | 1.5 | (0.9) | 33 | 1.9 | (1.1) |
| **SIENAX_Bl** | 16 | 1023.7 | (76.2) | 10 | 1125.0 | (100.9) | 8 | 1026.0 | (118.9) | 19 | 1042.8 | (71.7) | 34 | 1081.4 | (122.4) |
| **SIENAX_Rp** | 16 | 1026.2 | (129) | 10 | 1108.7 | (108.1) | 8 | 1002.6 | (86.4) | 19 | 1038.3 | (115) | 34 | 1056.4 | (115.6) |
| **SIENAX_BV** | 16 | 0.8 | (2.9) | 10 | 1.5 | (1.5) | 8 | 1.5 | (1.9) | 19 | 1.0 | (2.7) | 34 | 1.5 | (1.6) |
| **SIENAX_KBSI** | 16 | 1.6 | (1.0) | 10 | 1.6 | (1.2) | 8 | 3.2 | (1.6) | 19 | 1.7 | (1.0) | 34 | 2.1 | (1.2) |
| **SIENA_PBVC** | 16 | 2.2 | (1.2) | 10 | 2.3 | (1.1) | 8 | 4.4 | (2.4) | 19 | 2.4 | (1.2) | 34 | 2.9 | (1.8) |
| **SPM_Bl** | 16 | 1007.7 | (79) | 10 | 1063.4 | (134.7) | 8 | 994.0 | (114.8) | 19 | 1027.3 | (68.5) | 34 | 1039.6 | (124.7) |
| **SPM_Rp** | 16 | 964.5 | (95.3) | 10 | 1038.3 | (151.4) | 8 | 942.1 | (134.5) | 19 | 979.9 | (82.4) | 34 | 993.8 | (139.7) |
| **SPM_BV** | 16 | 2.1 | (1.7) | 10 | 2.6 | (3.0) | 8 | 4.2 | (3.7) | 19 | 2.4 | (1.6) | 34 | 3.1 | (2.5) |
| **SPM_KBSI** | 15 | 1.8 | (1.0) | 9 | 1.7 | (1.5) | 8 | 3.7 | (2.0) | 18 | 1.9 | (1.0) | 32 | 2.5 | (1.5) |
| **SPM_GBSI** | 15 | 2.0 | (1.1) | 10 | 1.9 | (1.4) | 8 | 3.9 | (2.1) | 18 | 2.1 | (1.1) | 33 | 2.6 | (1.6) |
| **Long_SPM_PBVC** | 16 | 1.0 | (0.9) | 10 | 1.2 | (0.8) | 8 | 0.6 | (1.2) | 19 | 1.0 | (0.8) | 34 | 0.9 | (0.9) |

**Table S4** Mean and standard deviation (sd) of the annualised rate of change in whole-brain volume (%) for controls and the clinical, genetic and pathology FTD subgroups in the refined (n = 226) cohort.

| **n=226** | **Controls (n=66)** | **Clinical subgroups** | | | | | **Genetic subgroups** | | | **Pathology subgroups** | |
| --- | --- | --- | --- | --- | --- | --- | --- | --- | --- | --- | --- |
|  |  | **bvFTD (n=56)** | **svPPA (n=38)** | **nfvPPA (n=42)** | **lvPPA (n=18)** | **PPA-NOS (n=6)** | ***MAPT* (n=14)** | ***C9orf72* (n=9)** | ***GRN* (n=8)** | **Tau (n=17)** | **TDP-43 (n=32)** |
| **BMAPS_BV** | 0.4 (0.8) | 2.2 (1.7) | 2.3 (1.2) | 2.7 (1.8) | 2.7 (1.6) | 2.9 (1.0) | 2.1 (1.0) | 1.9 (1.5) | 3.0 (2.6) | 2.3 (1.0) | 2.5 (1.6) |
| **BMAPS_KBSI** | 0.4 (0.7) | 2.0 (1.3) | 2.3 (0.9) | 2.6 (1.3) | 2.4 (0.9) | 2.7 (0.9) | 2.0 (1.0) | 1.8 (1.3) | 3.7 (1.8) | 2.1 (1.0) | 2.5 (1.4) |
| **Freesurfer_BV** | 0.6 (1.2) | 2.6 (3.9) | 1.6 (4.2) | 2.6 (3.0) | 1.3 (3.6) | 2.8 (1.0) | 1.6 (1.7) | 2.5 (3.1) | 3.7 (1.7) | 2.1 (2.0) | 2.9 (3.2) |
| **Freesurfer_KBSI** | 0.3 (0.5) | 1.7 (1.3) | 2.0 (1.0) | 2.4 (1.3) | 1.8 (1.4) | 2.6 (0.9) | 1.8 (0.9) | 1.6 (1.1) | 2.8 (2.0) | 1.9 (0.9) | 2.1 (1.4) |
| **GIF_BV** | 0.2 (0.6) | 1.1 (1.2) | 1.1 (0.7) | 1.4 (0.7) | 1.4 (1.7) | 1.8 (0.9) | 1.1 (0.6) | 1.3 (1.0) | 1.2 (1.8) | 1.2 (0.5) | 1.3 (1.1) |
| **GIF_KBSI** | 0.4 (0.7) | 1.7 (1.1) | 1.9 (0.7) | 2.2 (1.1) | 1.9 (1.2) | 2.4 (0.9) | 1.7 (0.8) | 1.5 (1.1) | 3.1 (1.5) | 1.8 (0.8) | 2.1 (1.2) |
| **GIF_GBSI** | 0.4 (0.7) | 1.7 (1.2) | 1.9 (0.7) | 2.2 (1.1) | 2.1 (0.8) | 2.4 (0.9) | 1.7 (0.8) | 1.5 (1.1) | 3.1 (1.4) | 1.8 (0.8) | 2.1 (1.2) |
| **MALP-EM_BV** | 0.2 (1.0) | 1.0 (2.5) | 0.4 (1.9) | 0.5 (1.8) | 1.8 (4.3) | 1.7 (0.7) | 0.5 (1.0) | 0.7 (1.7) | -0.7 (4.0) | 0.7 (1.1) | 0.4 (2.3) |
| **MALP-EM_KBSI** | 0.3 (0.4) | 1.4 (1.0) | 1.5 (0.6) | 1.8 (1.0) | 1.7 (0.6) | 2.0 (0.8) | 1.3 (0.7) | 1.2 (0.7) | 2.6 (1.1) | 1.3 (0.6) | 1.7 (0.9) |
| **MALP-EM_GBSI** | 0.3 (0.6) | 1.6 (1.1) | 1.8 (0.7) | 2.1 (1.1) | 1.7 (1.1) | 2.3 (0.8) | 1.5 (0.9) | 1.3 (0.9) | 3.0 (1.3) | 1.6 (0.8) | 1.9 (1.1) |
| **SIENAX_BV** | 0.2 (1.0) | 1.2 (2.4) | 1.6 (2.3) | 1.8 (1.8) | 1.7 (1.6) | 2.5 (1.1) | 0.8 (2.9) | 1.3 (1.5) | 1.5 (1.9) | 1.0 (2.7) | 1.5 (1.7) |
| **SIENAX_KBSI** | 0.4 (0.7) | 1.7 (1.1) | 1.9 (0.8) | 2.4 (1.2) | 1.9 (1.4) | 2.7 (0.9) | 1.7 (1.0) | 1.7 (1.2) | 3.2 (1.6) | 1.8 (0.9) | 2.2 (1.2) |
| **SIENA_PBVC** | 0.5 (0.6) | 2.6 (1.9) | 3.0 (1.0) | 3.3 (2.0) | 3.2 (1.3) | 3.2 (1.3) | 2.4 (1.2) | 2.1 (1.1) | 4.4 (2.4) | 2.5 (1.2) | 2.9 (1.8) |
| **SPM_BV** | 0.5 (1.0) | 2.8 (2.1) | 2.9 (1.8) | 3.2 (2.3) | 3.2 (2.7) | 3.2 (1.1) | 2.5 (1.4) | 2.1 (3.0) | 4.2 (3.7) | 2.7 (1.4) | 3.0 (2.5) |
| **SPM_KBSI** | 0.4 (0.6) | 2.0 (1.4) | 2.2 (1.0) | 2.7 (1.4) | 2.2 (1.4) | 2.7 (1.0) | 1.9 (0.9) | 1.7 (1.5) | 3.7 (2.0) | 2.0 (0.9) | 2.5 (1.5) |
| **SPM_GBSI** | 0.4 (0.7) | 2.2 (1.5) | 2.4 (0.9) | 2.8 (1.4) | 2.3 (1.5) | 2.8 (0.9) | 2.1 (1.0) | 1.7 (1.4) | 3.9 (2.1) | 2.2 (1.0) | 2.6 (1.6) |
| **Long_SPM_PBVC** | 0.2 (0.5) | 1.1 (0.9) | 0.9 (0.7) | 1.2 (1.0) | 2.6 (0.7) | 1.5 (0.6) | 1.1 (0.8) | 1.2 (0.8) | 0.6 (1.2) | 1.1 (0.7) | 0.9 (0.9) |

**Table S5** Effect size calculations with 95% BCa confidence intervals for clinical, genetic and pathology FTD subgroups *

| **Measure** | **Clinical Subgroups** | | | | | | | | | |
| --- | --- | --- | --- | --- | --- | --- | --- | --- | --- | --- |
|  | **bvFTD** | | **svPPA** | | **nfvPPA** | | **lvPPA** | | **PPA-NOS** | |
| **n=226** | **Effect Size** | **95% CI (BCa)** | **Effect Size** | **95% CI (BCa)** | **Effect Size** | **95% CI (BCa)** | **Effect Size** | **95% CI (BCa)** | **Effect Size** | **95% CI (BCa)** |
| **BMAPS_BV** | **1.1** | [0.8–1.4] | **1.7** | [1.2–2.1] | **1.5** | [1.1–2.0] | **1.3** | [0.9–1.8] | **2.4** | [1.6–3.9] |
| **BMAPS_KBSI** | **1.2** | [0.9–1.5] | **2.1** | [1.5–2.7] | **1.7** | [1.3–2.1] | **2.1** | [1.4–2.7] | **2.4** | [1.8–3.8] |
| **Freesurfer_BV** | 0.5 | [0.2–0.8] | 0.3 | [-0.2–0.8] | 0.6 | [0.2–1.0] | 0.2 | [-0.3–0.8] | **2.2** | [0.8–4.4] |
| **Freesurfer_KBSI** | **1.2** | [0.9–1.4] | **1.9** | [1.3–2.4] | **1.7** | [1.3–2.1] | **1.1** | [0.2–2.2] | **2.7** | [2.0–3.6] |
| **GIF_BV** | 0.8 | [0.5–1.1] | **1.5** | [0.7–2.1] | **1.7** | [1.2–2.3] | 0.7 | [-0.1–1.4] | **1.8** | [1.0–3.1] |
| **GIF_KBSI** | **1.2** | [0.9–1.5] | **2.2** | [1.6–2.7] | **1.7** | [1.2–2.1] | **1.2** | [0.1–2.4] | **2.3** | [1.7–4.1] |
| **GIF_GBSI** | **1.2** | [0.9–1.5] | **2.2** | [1.6–2.8] | **1.7** | [1.3–2.2] | **2.2** | [1.6–2.8] | **2.4** | [1.7–4.2] |
| **MALP-EM_BV** | 0.3 | [0.0–0.6] | 0.1 | [-0.3–0.6] | 0.2 | [-0.2–0.6] | 0.4 | [-0.2–0.6] | **2.1** | [1.0–3.9] |
| **MALP-EM_KBSI** | **1.1** | [0.9–1.4] | **2.3** | [1.6–2.9] | **1.6** | [1.1–2.0] | **2.2** | [1.7–2.7] | **2.3** | [1.7–3.6] |
| **MALP-EM_GBSI** | **1.2** | [0.9–1.5] | **2.2** | [1.6–2.7] | **1.7** | [1.2–2.1] | **1.2** | [0.2–2.3] | **2.4** | [1.5–4.1] |
| **SIENAX_BV** | 0.4 | [0.0–0.8] | 0.9 | [0.6–1.2] | 0.8 | [0.3–1.2] | 0.9 | [0.3–1.5] | **2.0** | [1.0–4.1] |
| **SIENAX_KBSI** | **1.1** | [0.7–1.4] | **2.1** | [1.4–2.7] | **1.7** | [1.2–2.0] | **1.1** | [0.1–2.0] | **2.6** | [1.6–4.5] |
| **SIENA_PBVC** | **1.1** | [0.9–1.4] | **2.5** | [2.0–3.0] | **1.5** | [1.1–2.0] | **2.2** | [1.5–3.0] | **2.1** | [1.6–3.2] |
| **SPM_BV** | **1.1** | [0.9–1.4] | **1.9** | [1.4–2.5] | **1.2** | [0.7–1.6] | **1.1** | [0.3–1.8] | **2.5** | [1.5–3.9] |
| **SPM_KBSI** | **1.1** | [0.9–1.4] | **2.0** | [1.5–2.6] | **1.6** | [1.1–2.1] | **1.2** | [0.2–2.4] | **2.1** | [1.7–3.4] |
| **SPM_GBSI** | **1.2** | [0.9–1.5] | **2.3** | [1.7–2.9] | **1.7** | [1.2–2.2] | **1.2** | [0.2–2.3] | **2.6** | [2.0–4.1] |
| **Long_SPM_PBVC** | **1.0** | [0.7–1.3] | **1.0** | [0.–1.5] | **1.1** | [0.4–1.5] | **1.9** | [1.2–2.5] | **2.1** | [1.3–3.2] |

* Effect sizes of one or above are in **bold** to indicate results demonstrate a high degree of group separation from controls for that subgroup and measure

**Table S5 continued** Effect size calculations with 95% BCa confidence intervals for clinical, genetic and pathology FTD subgroups *

| **Measure** | **Genetic Subgroups** | | | | | | **Pathology Subgroups** | | | |
| --- | --- | --- | --- | --- | --- | --- | --- | --- | --- | --- |
|  | ***MAPT*** | | ***C9orf72*** | | ***GRN*** | | **Tau** | | **TDP-43** | |
| **n=226** | **Effect Size** | **95% CI (BCa)** | **Effect Size** | **95% CI (BCa)** | **Effect Size** | **95% CI (BCa)** | **Effect Size** | **95% CI (BCa)** | **Effect Size** | **95% CI (BCa)** |
| **BMAPS_BV** | **1.6** | [1.1–2.3] | **1.2** | [0.6–1.9] | **1.0** | [0.4–1.6] | **1.8** | [1.2–2.5] | **1.3** | [0.8–1.7] |
| **BMAPS_KBSI** | **1.7** | [1.1–2.5] | **1.2** | [0.5–2.0] | **1.8** | [1.0–2.6] | **1.7** | [1.2–2.5] | **1.5** | [1.1–2.0] |
| **Freesurfer_BV** | 0.8 | [-0.1–1.7] | **1.2** | [0.4–2.0] | **1.8** | [1.0–2.9] | 0.8 | [0.2–1.2] | 0.7 | [0.3–1.1] |
| **Freesurfer_KBSI** | **1.8** | [1.2–2.7] | **1.1** | [0.4–1.7] | **1.2** | [0.5–2.4] | **1.8** | [1.3–2.5] | **1.3** | [0.9–1.6] |
| **GIF_BV** | **1.8** | [1.2–2.4] | **1.1** | [0.4–2.2] | 0.6 | [-0.4–2.6] | **2.0** | [1.3–2.7] | **1.0** | [0.4–1.5] |
| **GIF_KBSI** | **1.7** | [1.2–2.6] | **1.1** | [0.3–1.8] | **1.9** | [1.1–2.8] | **1.8** | [1.2–2.6] | **1.5** | [1.1–1.9] |
| **GIF_GBSI** | **1.8** | [1.2–2.7] | **1.1** | [0.4–1.8] | **1.9** | [1.0–2.9] | **1.9** | [1.3–2.7] | **1.5** | [1.1–2.0] |
| **MALP-EM_BV** | 0.2 | [-0.5–1.1] | 0.3 | [-0.8–1.6] | -0.2 | [-1.1–0.8] | 0.4 | [-0.2–1.0] | 0.1 | [-0.3–0.5] |
| **MALP-EM_KBSI** | **1.5** | [0.9–2.2] | **1.2** | [0.6–1.8] | **2.2** | [1.3–3.3] | **1.6** | [1.0–2.3] | **1.5** | [1.1–1.9] |
| **MALP-EM_GBSI** | **1.5** | [0.8–2.3] | **1.1** | [0.6–1.7] | **2.1** | [1.2–3.2] | **1.6** | [0.9–2.3] | **1.5** | [1.1–2.0] |
| **SIENAX_BV** | 0.2 | [-0.5–1.7] | 0.7 | [-0.3–1.6] | 0.7 | [-0.3–1.8] | 0.3 | [-0.4–1.8] | 0.8 | [0.4–1.2] |
| **SIENAX_KBSI** | **1.5** | [1.0–2.0] | **1.1** | [0.3–1.8] | **1.8** | [0.8–3.1] | **1.5** | [1.0–2.0] | **1.4** | [1.0–1.8] |
| **SIENA_PBVC** | **1.6** | [0.7–2.4] | **1.5** | [0.7–2.6] | **1.6** | [0.8–2.4] | **1.7** | [0.8–2.6] | **1.4** | [0.9–1.8] |
| **SPM_BV** | **1.4** | [0.7–2.2] | 0.6 | [0.0–1.2] | **1.0** | [0.6–1.4] | **1.6** | [0.9–2.5] | **1.0** | [0.6–1.4] |
| **SPM_KBSI** | **1.6** | [1.1–2.2] | 0.9 | [0.3–1.5] | **1.6** | [1.0–2.3] | **1.7** | [1.2–2.3] | **1.3** | [0.9–1.8] |
| **SPM_GBSI** | **1.7** | [1.1–2.5] | **1.0** | [0.4–1.6] | **1.6** | [1.0–2.3] | **1.8** | [1.2–2.6] | **1.4** | [1.0–1.8] |
| **Long_SPM_PBVC** | **1.2** | [0.6–1.9] | **1.2** | [0.4–2.0] | 0.3 | [-0.6–1.7] | **1.3** | [0.6–2.0] | 0.8 | [0.2–1.3] |

* Effect sizes of one or above are in **bold** to indicate results demonstrate a high degree of group separation from controls for that subgroup and measure

**Table S6** Linear regression analysis results reporting the difference in mean compared to controls [95% CI] for clinical, genetic and pathology FTD subgroups for all longitudinal annual whole-brain measures of change. *

| **n = 226** | **Clinical Subgroups** | | | | | | | | | |
| --- | --- | --- | --- | --- | --- | --- | --- | --- | --- | --- |
|  | **bvFTD** | | **svPPA** | | **nfvPPA** | | **lvPPA** | | **PPA-NOS** | |
| **Measure** | **Coef. [95% CI]** | ***p*-value** | **Coef. [95% CI]** | ***p*-value** | **Coef. [95% CI]** | ***p*-value** | **Coef. [95% CI]** | ***p*-value** | **Coef. [95% CI]** | ***p*-value** |
| **BMAPS_BV** | 1.8 [1.3–2.3] | **<0.001** | 1.9 [1.4–2.4] | **<0.001** | 2.3 [1.8–2.8] | **<0.001** | 2.4 [1.7–3.1] | **<0.001** | 2.5 [1.4–3.6] | **<0.001** |
| **BMAPS_KBSI** | 1.6 [1.2–2.0] | **<0.001** | 1.8 [1.4–2.3] | **<0.001** | 2.2 [1.8–2.7] | **<0.001** | 2.0 [1.4–2.6] | **<0.001** | 2.3 [1.4–3.2] | **<0.001** |
| **Freesurfer_BV** | 1.9 [0.8–3.0] | *0.001* | 1.2 [-0.1–2.4] | 0.062 | 2.0 [0.8–3.1] | *0.001* | 0.4 [-1.2–2.1] | 0.600 | 2.0 [-0.6–4.5] | 0.129 |
| **Freesurfer_KBSI** | 1.5 [1.1–1.8] | **<0.001** | 1.7 [1.3–2.1] | **<0.001** | 2.2 [1.8–2.6] | **<0.001** | 1.6 [1.0–2.1] | **<0.001** | 2.3 [1.4–3.1] | **<0.001** |
| **GIF_BV** | 0.9 [0.5–1.2] | **<0.001** | 0.9 [0.6–1.3] | **<0.001** | 1.2 [0.8–1.5] | **<0.001** | 1.2 [0.7–1.7] | **<0.001** | 1.6 [0.8–2.3] | **<0.001** |
| **GIF_KBSI** | 1.3 [1.0–1.7] | **<0.001** | 1.5 [1.2–1.9] | **<0.001** | 1.8 [1.5–2.2] | **<0.001** | 1.5 [0.9–2.0] | **<0.001** | 2.0 [1.2–2.8] | **<0.001** |
| **GIF_GBSI** | 1.4 [1.0–1.7] | **<0.001** | 1.6 [1.2–1.9] | **<0.001** | 1.9 [1.5–2.2] | **<0.001** | 1.7 [1.2–2.2] | **<0.001** | 2.0 [1.3–2.8] | **<0.001** |
| **MALP-EM_BV** | 0.7 [-0.1–1.5] | 0.069 | 0.1 [-0.7–1.0] | 0.778 | 0.3 [-0.5–1.2] | 0.455 | 1.6 [0.4–2.8] | *0.007* | 1.5 [-0.3–3.3] | 0.112 |
| **MALP-EM_KBSI** | 1.2 [0.9–1.4] | **<0.001** | 1.3 [1.0–1.6] | **<0.001** | 1.5 [1.2–1.8] | **<0.001** | 1.5 [1.1–1.9] | **<0.001** | 1.8 [1.1–2.4] | **<0.001** |
| **MALP-EM_GBSI** | 1.4 [1.0–1.7] | **<0.001** | 1.5 [1.1–1.9] | **<0.001** | 1.8 [1.4–2.1] | **<0.001** | 1.4 [0.9–1.9] | **<0.001** | 2.0 [1.2–2.7] | **<0.001** |
| **SIENAX_BV** | 1.0 [0.4–1.7] | *0.002* | 1.4 [0.7–2.1] | **<0.001** | 1.6 [0.9–2.3] | **<0.001** | 1.5 [0.5–2.4] | *0.002* | 2.3 [0.8–3.7] | *0.003* |
| **SIENAX_KBSI** | 1.2 [0.9–1.6] | **<0.001** | 1.6 [1.1–2.0] | **<0.001** | 2.0 [1.6–2.4] | **<0.001** | 1.5 [0.9–2.0] | **<0.001** | 2.2 [1.4–3.1] | **<0.001** |
| **SIENA_PBVC** | 2.2 [1.7–2.5] | **<0.001** | 2.5 [1.9–3.1] | **<0.001** | 2.9 [2.3–3.5] | **<0.001** | 2.9 [2.1–3.6] | **<0.001** | 2.8 [1.6–4.0] | **<0.001** |
| **SPM_BV** | 2.4 [1.7–3.0] | **<0.001** | 2.5 [1.8–3.2] | **<0.001** | 2.7 [2.0–3.4] | **<0.001** | 2.7 [1.8–3.7] | **<0.001** | 2.7 [1.2–4.2] | *0.001* |
| **SPM_KBSI** | 1.6 [1.1–2.0] | **<0.001** | 1.8 [1.3–2.2] | **<0.001** | 2.2 [1.8–2.7] | **<0.001** | 1.8 [1.2–2.4] | **<0.001** | 2.3 [1.3–3.2] | **<0.001** |
| **SPM_GBSI** | 1.8 [1.4–2.3] | **<0.001** | 2.0 [1.5–2.5] | **<0.001** | 2.4 [1.9–2.9] | **<0.001** | 2.0 [1.3–2.6] | **<0.001** | 2.4 [1.4–3.4] | **<0.001** |
| **Long_SPM_PBVC** | 0.9 [0.6–1.1] | **<0.001** | 0.7 [0.4–1.0] | **<0.001** | 1.0 [0.7–1.3] | **<0.001** | 1.2 [0.6–1.9] | **<0.001** | 1.2 [0.6–1.9] | **<0.001** |

* All analyses adjusted for age, sex and scanner type: ***p* < 0.001 bolded**, *p < 0.05 italicised*

**Table S6 continued** Linear regression analysis results reporting the difference in mean compared to controls [95% CI] for clinical, genetic and pathology FTD subgroups for all longitudinal annual whole-brain measures of change. *

| **n = 226** | **Genetic Subgroups** | | | | | | **Pathology Subgroups** | | | |
| --- | --- | --- | --- | --- | --- | --- | --- | --- | --- | --- |
|  | ***MAPT*** | | ***C9ORF72*** | | ***GRN*** | | **Tau** | | **TDP-43** | |
| **Measure** | **Coef. [95% CI]** | ***p*-value** | **Coef. [95% CI]** | ***p*-value** | **Coef. [95% CI]** | ***p*-value** | **Coef. [95% CI]** | ***p*-value** | **Coef. [95% CI]** | ***p*-value** |
| **BMAPS_BV** | 1.8 [1.2–2.5] | **<0.001** | 1.4 [0.5–2.2] | 0.001 | 2.7 [1.8–3.5] | **<0.001** | 2.0 [1.4–2.7] | **<0.001** | 2.1 [1.6–2.6] | **<0.001** |
| **BMAPS_KBSI** | 1.6 [1.1–2.2] | **<0.001** | 1.3 [0.6–1.9] | **<0.001** | 3.3 [2.6–4.0] | **<0.001** | 1.8 [1.2–2.3] | **<0.001** | 2.1 [1.7–2.5] | **<0.001** |
| **Freesurfer_BV** | 1.2 [0.4–1.9] | 0.002 | 1.6 [0.7–2.5] | *0.001* | 3.3 [2.4–4.2] | **<0.001** | 1.7 [0.6–2.8] | *0.003* | 2.4 [1.5–3.3] | **<0.001** |
| **Freesurfer_KBSI** | 1.6 [1.1–2.1] | **<0.001** | 1.3 [0.6–1.9] | **<0.001** | 2.5 [1.9–3.1] | **<0.001** | 1.6 [1.1–2.1] | **<0.001** | 1.8 [1.4–2.2] | **<0.001** |
| **GIF_BV** | 1.0 [0.5–1.5] | **<0.001** | 1.0 [0.4–1.6] | *0.001* | 1.1 [0.5–1.6] | *0.001* | 1.0 [0.6–1.4] | **<0.001** | 1.1 [0.7–1.4] | **<0.001** |
| **GIF_KBSI** | 1.4 [0.9–1.9] | **<0.001** | 1.0 [0.4–1.6] | *0.001* | 2.8 [2.2–3.4] | **<0.001** | 1.5 [1.0–2.0] | **<0.001** | 1.8 [1.4–2.1] | **<0.001** |
| **GIF_GBSI** | 1.4 [0.9–1.9] | **<0.001** | 1.0 [0.5–1.7] | **<0.001** | 2.8 [2.2–3.4] | **<0.001** | 1.5 [1.0–2.0] | **<0.001** | 1.8 [1.4–2.1] | **<0.001** |
| **MALP-EM_BV** | 0.3 [-0.6–1.2] | 0.483 | 0.3 [-0.8–1.5] | 0.543 | -1.0 [-2.1–0.1] | 0.084 | 0.4 [-0.4–1.3] | 0.296 | 0.1 [-0.6–0.7] | 0.870 |
| **MALP-EM_KBSI** | 1.1 [0.7–1.4] | **<0.001** | 0.9 [0.5–1.4] | **<0.001** | 2.4 [2.0–2.8] | **<0.001** | 1.1 [0.8–1.5] | **<0.001** | 1.4 [1.2–1.7] | **<0.001** |
| **MALP-EM_GBSI** | 1.3 [0.8–1.7] | **<0.001** | 1.0 [0.4–1.5] | *0.001* | 2.7 [2.2–3.3] | **<0.001** | 1.4 [0.9–1.8] | **<0.001** | 1.6 [1.3–2.0] | **<0.001** |
| **SIENAX_BV** | 0.5 [-0.5–1.4] | 0.320 | 0.7 [-0.5–1.8] | 0.244 | 1.4 [0.3–2.5] | *0.015* | 0.8 [-0.1–1.6] | 0.079 | 1.2 [0.6–1.9] | **<0.001** |
| **SIENAX_KBSI** | 1.4 [0.9–1.9] | **<0.001** | 1.1 [0.5–1.8] | **<0.001** | 2.9 [2.2–3.5] | **<0.001** | 1.5 [1.0–2.0] | **<0.001** | 1.7 [1.4–2.1] | **<0.001** |
| **SIENA_PBVC** | 2.1 [1.5–2.4] | **<0.001** | 1.6 [0.9–2.4] | **<0.001** | 3.9 [3.1–4.7] | **<0.001** | 2.2 [1.6–2.9] | **<0.001** | 2.5 [2.0–2.9] | **<0.001** |
| **SPM_BV** | 2.2 [1.2–3.2] | **<0.001** | 1.6 [0.4–2.8] | *0.010* | 3.7 [2.5–5.0] | **<0.001** | 2.4 [1.5–3.3] | **<0.001** | 2.6 [1.9–3.3] | **<0.001** |
| **SPM_KBSI** | 1.6 [1.0–2.2] | **<0.001** | 1.3 [0.6–2.0] | *0.001* | 3.3 [2.6–4.0] | **<0.001** | 1.7 [1.1–2.3] | **<0.001** | 2.1 [1.6–2.5] | **<0.001** |
| **SPM_GBSI** | 1.8 [1.2–2.4] | **<0.001** | 1.3 [0.5–2.0] | *0.001* | 3.5 [2.8–4.3] | **<0.001** | 1.9 [1.3–2.5] | **<0.001** | 2.2 [1.7–2.7] | **<0.001** |
| **Long_SPM_PBVC** | 1.0 [0.6–1.4] | **<0.001** | 0.8 [0.3–1.3] | *0.001* | 0.5 [0.0–0.9] | 0.056 | 1.0 [0.6–1.3] | **<0.001** | 0.7 [0.4–0.9] | **<0.001** |

* All analyses adjusted for age, sex and scanner type: ***p* < 0.001 bolded**, *p < 0.05 italicised*

**Table S7** summarises segmentation and registration failures for each of the six automated segmentation techniques. All SIENA outputs and reports were visually reviewed and passed. The longitudinal SPM deformation fields for the 262 pairs were all visually assessed and found to be applied to the correct regions with no gross errors evident.

**Table S7** Overall numbers of segmentations and registrations that failed for each of the six segmentation methods due to incomplete pipeline execution or QC issue

|  | **Segmentation** | | **KBSI** | | **GBSI** | |
| --- | --- | --- | --- | --- | --- | --- |
|  | Pipeline failed to complete | QC Fail | Pipeline failed to complete | QC Fail | Pipeline failed to complete | QC Fail |
| **SPM** | 1 Rp | 0 | 4 | 10 | 4 | 9 |
| **BMAPS** | 0 | 0 | 0 | 13 | NA | NA |
| **MALP-EM** | 0 | 0 | 2 | 8 | 1 | 12 |
| **SIENAX** | 0 | 0 | 1 | 4 | NA | NA |
| **Freesurfer** | 0 | 1 Bl, 3 Rp | 0 | 5 | NA | NA |
| **GIF** | 0 | 0 | 0 | 10 | 0 | 12 |

* Bl = baseline image, Rp = repeat image.

**Table S8** Breakdown of scanner information slit by scanning period and displaying manufacturer, field strength and key acquisition parameters.

| **Acquisition start (yr)** | **Scanner strength** | **Scanner manufacturer** | **Key acquisition parameters*** | | | | |
| --- | --- | --- | --- | --- | --- | --- | --- |
|  |  |  | **Sequence name** | **TI (ms)** | **TE (ms)** | **TR (ms)** | **Voxel size** |
| 1992–1999 | 1.5 Tesla | GE Signa | spoiled gradient echo | 650 | 5 | 12 | 1.5×1.5×1.5 mm^3^ |
| 2000–2004 | 1.5 Tesla | GE Signa | inversion recovery-prepared spoiled GRASS sequence  (IR-SPGR) | 650 | 5 | 12 | 1.5×1.5×1.5 mm^3^ |
| 2005–2008 | 1.5 Tesla | GE Signa | spoiled gradient echo | 650 | 5 | 12 | 1.5×1.5×1.5 mm^3^ |
| 2009–2014 | 3 Tesla | Siemens Tim Trio (using a 32 channel coil) | magnetization-prepared rapid gradient echo (MPRAGE) | 900 | 2.9 | 2200 | 1.1×1.1×1.1 mm^3^ |
| 2015–2018 | 3 Tesla | Siemens MAGNETOM Prisma (using 64 channel coil) | MPRAGE sagittal | 850 | 2.93 | 2000 | 1.1×1.1×1.1 mm^3^ |

* TI = Inversion Time, TE = Echo Time and TR = Repetition Ti
